# Forced Convection Nanofluid Heat Transfer as a Function of Distance in Microchannels

**DOI:** 10.3390/ma14113021

**Published:** 2021-06-02

**Authors:** Saeid Vafaei, Jonathan A. Yeager, Peter Daluga, Branden Scherer

**Affiliations:** Department of Mechanical Engineering, Bradley University, Peoria, IL 61625, USA; jyeager@mail.bradley.edu (J.A.Y.); pdaluga@mail.bradley.edu (P.D.); bscherer@mail.bradley.edu (B.S.)

**Keywords:** heat transfer coefficient, nanofluid, nanoparticle, laminar, turbulent

## Abstract

As electronic devices become smaller and more powerful, the demand for micro-scale thermal management becomes necessary in achieving a more compact design. One way to do that is enhancing the forced convection heat transfer by adding nanoparticles into the base liquid. In this study, the nanofluid forced convection heat transfer coefficient was measured inside stainless-steel microchannels (ID = 210 μm) and heat transfer coefficient as a function of distance was measured to explore the effects of base liquid, crystal phase, nanoparticle material, and size on heat transfer coefficient. It was found that crystal phase, characteristics of nanoparticles, the thermal conductivity and viscosity of nanofluid can play a significant role on heat transfer coefficient. In addition, the effects of man-made and commercial TiO_2_ on heat transfer coefficient were investigated and it was found that man-made anatase TiO_2_ nanoparticles were more effective to enhance the heat transfer coefficient, for given conditions. This study also conducted a brief literature review on nanofluid forced convection heat transfer to investigate how nanofluid heat transfer coefficient as a function of distance would be affected by effective parameters such as base liquid, flow regime, concentration, and the characteristics of nanoparticles (material and size).

## 1. Introduction

Over the last century, the researchers have tried to enhance the forced convection heat transfer coefficient in the macroscale. Indeed, forced convection heat transfer plays a crucial role in a number of applications such as power generation, chemical processing, transportation, microelectronics, etc. The rapid growth of technology and the increasing demand of industries for high heat transfer rates have motivated researchers to find novel ways to enhance the heat transfer coefficient, such as surface modification, geometry enhancement, changing the flow regime and possibly applying electric or magnetic fields [1,2,3,4,5]. Recently, power enhancements and the miniaturization of devices have driven researchers to enhance the thermal management of devices in microscale. These more compact technologies also require the use of smaller channels. Many suggestions on how to classify these smaller channels have been put forward. For instance, Kandlikar [6] proposed that conventional channels refer to any channel larger than 3 mm. Minichannels would then have diameters between 3 mm and 200 μm, and the smallest type of channel, microchannels, would have diameters under 200 μm and above 10 μm. Many efforts have been made to improve the heating and cooling of micro-systems, including the modification of the thermal properties of working fluids. The thermal properties of most working fluids are not good enough for high heat flux applications. The thermal physical properties of working fluids can be modified, by adding nanoparticles into the base liquid which is called nanofluid. Practically speaking, the thermal conductivity of most base liquids is relatively low [7] and the thermal conductivity of working fluids has a significant impact on forced convection heat transfer coefficient. It was observed that adding nanoparticles into the base liquid would increase the overall thermal conductivity of the mixture [1]. The mixture of pure liquid and nanoparticles is called nanofluid. Later on, it was observed that adding nanoparticles has great potential to enhance the forced convection heat transfer coefficient, because of thermal conductivity enhancement and energy transportation inside the nanofluid [2,3]. It was also observed that the forced convection heat transfer coefficient of nanofluids inside microchannels, along with other physical properties, depend on channel geometry, channel size (diameter and length), flow regime, base liquid, surfactant and homogeneity of nanofluids, concentration, and characteristics of nanoparticles such as size, material, shape, and coating. The effect of nanoparticles on the nanofluid heat transfer coefficient still is under investigation, and many researchers are working to understand how characteristics of nanoparticles can enhance the nanofluid heat transfer coefficient. To achieve the best nanofluid for given conditions, it is necessary to engineer the characteristics of the nanoparticles, the base liquid, and any possible surfactants. Recent investigations [8] indicated that adding nanoparticles would increase thermal conductivity and viscosity of working fluids, as results optimization of effects of nanoparticles on thermal conductivity and viscosity of nanofluids is necessary.

Initially, the micron-sized particles were used to enhance the thermal physical properties of the base liquids. The engineering applicability of micron-sized particle colloids was generally hindered by sedimentation, clogging, and poor suspension stability. The micrometric suspensions were unstable mainly because of gravity so nanoparticles, with or without coatings, were introduced to enhance the stability and avoid the agglomeration. Unlike the micrometric suspensions, nanofluids exhibit a good stability with a lower clogging possibility in microscale channels. Nanofluids that are colloidal dispersions of nanoparticles in a base fluid have been shown to maintain their stability with the use of surfactants and coating techniques. In most cases, nano-sized particles have remained stable in liquid for long periods of time. Indeed, nanofluids have been proposed as a promising candidate for thermal management of powerful devices, ranging from microscale to macroscale applications in a variety of key engineering applications including the thermal management of electronic devices in and out of space, powerful lasers, transportation systems, solar-liquid heating collectors, and many more. In general, the demand for more compact technology is steadily increasing nowadays and causing heat transfer systems to be smaller and more powerful.

In the case of solar collectors, the performance can be enhanced by improving thermal properties and absorption rate of working fluids which can be achieved by adding nanoparticles into the base liquid. Recently, nanofluids have received great attention because of their capability to enhance the heat transfer rate [4] and efficiency of solar collectors, simultaneously [5]. The recent review papers [9,10] indicated that nanofluids have great potential for applications in solar systems such as solar collectors [11], photovoltaic thermal systems [12], and thermal energy storage systems [13]. The efficiency of flat-plate collectors was reported to enhance 28.3%, using Al_2_O_3_–water nanofluid [5].

The purpose of this study is to understand the effects of nanoparticle material and base liquid on nanofluid heat transfer coefficient as a function of distance. This paper will study nanofluid heat transfer and mainly attempt to explain the effects of Reynolds number, base liquid, concentration, and characteristics of nanoparticles on heat transfer coefficient inside macro and micro channels, and responsible mechanisms of nanofluid forced convection heat transfer coefficient.

### 1.1. Effects of Nanoparticle Concentration

**Figure 1:**
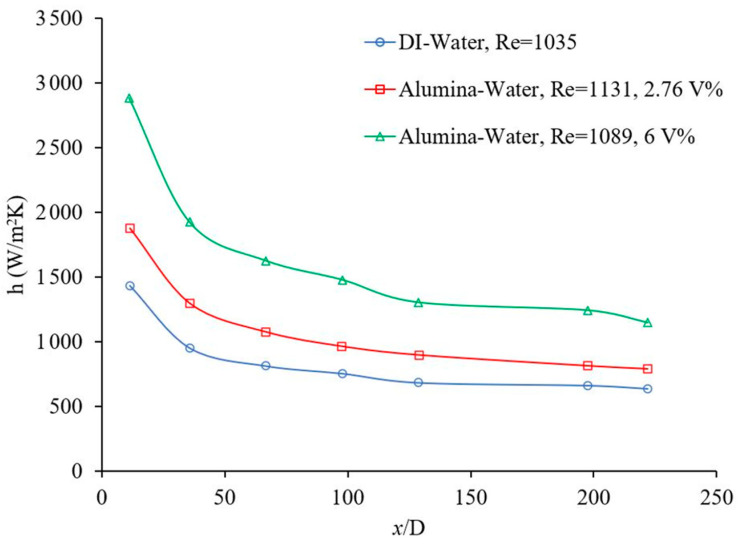
Effect of volume concentration on heat transfer coefficient, Rea et al. [14].

**Figure 2:**
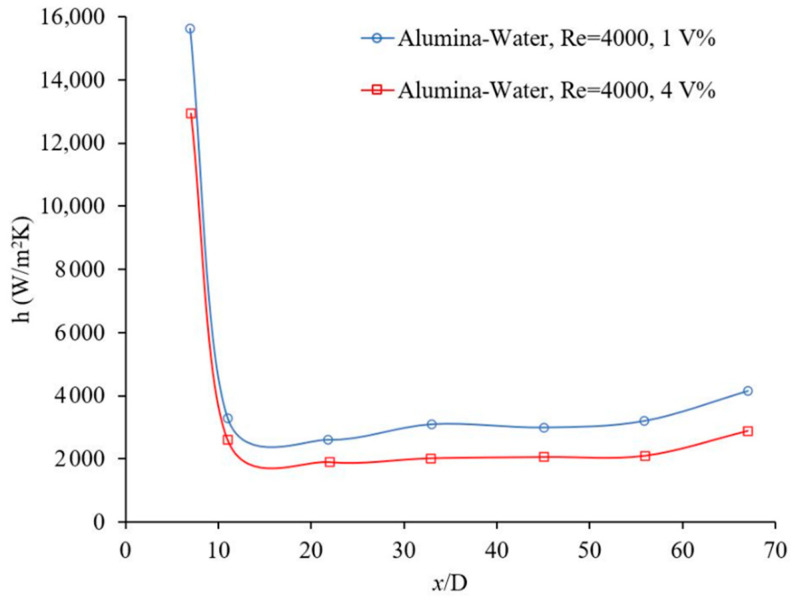
Effect of volume concentration on heat transfer coefficient, Sahin et al. [21].

Nanoparticle concentration can have a significant impact on forced convection heat transfer. This is illustrated in Figure 1 and Figure 2 which show the effects of alumina—water nanofluid concentration on forced convection heat transfer as a function of distance. x/D is the ratio of distance from starting heating point to inner diameter of channel. Figure 1 shows that the forced convection heat transfer increases with increasing the concentration of nanoparticles, for given conditions [14]. Similar results were observed by other researchers [4,15,16,17,18,19,20] as well. In contrast, Figure 2 shows that the forced convection heat transfer decreases with increasing concentration of nanoparticles and a similar trend was also observed by other researchers, for given conditions [21]. The concentration of nanoparticle would increase the thermal conductivity and viscosity of nanofluids. If thermal conductivity enhancement is dominated, the heat transfer coefficient would increase; and if viscosity enhancement is dominated, the heat transfer coefficient would decrease.

Rea et al. [14] conducted an experiment to show laminar forced convective heat transfer, using 50 nm alumina–water and zirconia–water nanofluids, and it was concluded that local nanofluid heat transfer coefficient can be predicted by conventional correlations under certain conditions. The inner diameter, outer diameter and length of the stainless-steel circular channel were 4.5 mm, 6.4 mm, and 1010 mm, respectively. It was found that the heat transfer coefficients in the entrance region and in the fully developed region were enhanced by 17% and 27%, respectively, for alumina–water nanofluid at φ = 6 vol % with respect to pure water. The zirconia–water nanofluid heat transfer coefficient was enhanced by approximately 2% in the entrance region and 3% in the fully developed region at φ = 1.32 vol %. The local heat transfer coefficient enhancement was affected in the fully developed region more than in the entrance region. Furthermore, the experimental Nusselt number was compared with theoretical predictions for deionized water and nanofluid working fluids and were in a good agreement with the theoretical predictions. Therefore, it was suggested that (a) the nanofluids can be treated as homogeneous mixtures and (b) the heat transfer coefficient enhancement was because of the effects of nanoparticles on the physical properties of the nanofluids [14]. In this research, the nanofluid behaved as a uniform working fluid because (a) the nanoparticles and base liquid were mixed homogeneously at the molecular level and (b) the measured physical properties were in a good agreement with the physical properties in working circumstances. Detailed information on characterization of these nanofluids can be found in [22].

Similarly, the variation of the Nusselt number as a function of axial distance for the base liquid and 1 wt % ZnO-water nanofluid (wt % is mass percent) at Re = 800 was measured and compared with prediction of the Shah [23] and Gnielinski [24] correlations where the pipe diameter was 8 mm. It was found there was a good agreement between the experimental Nusselt number and the predictions of the Shah and Gnielinski equations for the base liquid and the 1 wt % ZnO-water nanofluid. 1 wt % nanofluids were prepared by mixing zinc oxide, ZnO (40–100 nm), nanoparticles with a mixture of ethylene glycol and water, at 50/50% in volume [25]. Moreover, the effects of the volume fraction of three different nanoparticles (30 nm CuO, 30 nm TiO_2_, and 50 nm Al_2_O_3_) on turbine oil nanofluids was investigated inside a pipe in diameter of 7 mm in laminar flow regime [3]. The deviation from the Sieder-Tate correlation was within ±7% for both turbine oil and distilled water. The Nusselt number as a function of Reynolds number for different turbine oil nanofluids was also measured and compared with the prediction of the Sieder-Tate correlation [2]. The nanofluid heat transfer coefficient was observed to be much higher than the prediction of the Sieder-Tate correlation. It was concluded that the thermal conductivity enhancement of nanofluids is not the only responsible factor for the modification of the heat transfer coefficient and there must be different mechanisms to be involved, such as enhancement of the temperature gradient, particle migration, clustering effects due to non-uniform shear rate across the pipe, particle interactions, stochastic movements, and dispersion effects [3]. Since the nanoparticle volume fractions were relatively low, thermal conductivity enhancement might not be the main reason for the heat transfer coefficient enhancement; perhaps the chaotic movement of nanoparticles inside the nanofluid might have been the main reason responsible for the heat transfer coefficient enhancement. The chaotic movement of nanoparticles would affect the nanofluid forced convection heat transfer and physical properties and thus, the nanofluid physical properties should be measured in working conditions. The variation of the local heat transfer coefficient with axial distance was investigated for all three types of nanofluids with various nanoparticle volume fractions in the entrance region at Re = 750 and was found to increase for all three with nanoparticle volume fraction along the tube. The local heat transfer coefficient was more affected by nanoparticles at the entrance region and the effects of nanoparticles decreased with axial distance. Perhaps the temperature gradient was changed by the presence of nanoparticles inside the fluid and, as a result, the thermal boundary layer thickness decreased. This would consequently cause the heat transfer coefficient to increase. The heat transfer coefficient is proportional to the thermal conductivity and inversely proportional to the thermal boundary layer thickness. Moreover, the variation of the Nusselt number as a function of Reynolds number was investigated for all three types of nanofluids and for four different nanoparticle volume fractions. The nanoparticle volume fraction had a positive impact on the Nusselt number ratio $\left(Nu_{nf}/Nu_{oil}\right)$. The highest Nusselt number ratios of CuO-turbine oil, TiO_2_-turbine oil, and Al_2_O_3_-turbine oil at Re=800 were 1.38, 1.31, and 1.15, respectively. CuO-turbine oil nanofluid showed the highest heat transfer coefficient at 0.5 vol % nanoparticle volume fraction [3]. Similarly, the local heat transfer coefficient was measured [15] as a function of axial distance from the entrance for different volume fractions and different Reynolds numbers in the laminar flow regime (1057<Re<2070) where the inlet temperature was 30 °C. The alumina nanoparticle size was 20 nm and the pipe diameter was 11.1 mm. The Shah equation could not predict the nanofluid Nusselt number as a function of axial distance. It was found that (a) the local nanofluid heat transfer coefficient increased with volume fraction. For instance, the local nanofluid heat transfer coefficient was 18% higher than that of pure water, when the nanoparticle volume fraction was 0.9 vol %, x/D=47.74 and Reynolds number was ~1020 and (c) the maximum heat transfer coefficient enhancement was 23% when the volume fraction was 0.9 vol %, x/D=47.74 and the Reynolds number was ~2070. It was explained that the local nanofluid heat transfer coefficient enhancement was achieved because of nanofluid thermal conductivity enhancement [15]. A comparison between gold and silver nanofluid heat transfer coefficients was investigated inside a stainless-steel pipe in diameter of 2.27 mm for laminar flow, under the same heat flux [26]. The Nusselt number as a function of axial location was measured for two different volume flow rates (20 mL/min and 30 mL/min) for deionized water and compared with the prediction of Shah correlation [23]. A reasonable agreement was observed between experimental the Nusselt number and the Shah prediction for deionized water. The prediction of the Nusselt number was slightly more than the experimental results. Similar agreement was not observed between the nanofluid experimental data and the Shah prediction. The nanofluid local heat transfer coefficient and Nusselt number as a function axial distance was measured for different nanoparticle volume fractions and for different Reynolds numbers. The effects of the entrance region were noticed for all cases. It was observed that (a) the local heat transfer coefficient and Nusselt number increased with the nanoparticle volume fractions consistently and (b) the gold nanoparticles enhanced the heat transfer coefficient more than silver nanoparticles. The gold nanofluid heat transfer coefficient enhancement was 19% and 29% at x/D=8.81 and Re=400 where the nanoparticle volume fraction was 0.045% and 0.0667%, respectively. The Silver nanofluid heat transfer coefficient enhancement was 12% and 20% for the same conditions. The nanofluid heat transfer coefficient was enhanced because of (a) nanofluid thermal conductivity enhancement, (b) a reduction of the thermal boundary layer thickness, and (c) random motion of nanoparticles. The experimental results indicated that the heat transfer coefficient of gold nanofluid was higher than silver nanofluid heat transfer coefficient due to high thermal conductivity of gold nanofluid for the given conditions [26]. Numerical analysis was also used by Alsabery et al. [27] to model the flow of Al_2_O_3_ nanofluid through a wavy channel. It was found that heat transfer in the model increased as the Reynold’s number increased. Heat transfer was also found to be increased by making the channel wavier. This increased the mixing of the fluid and as such increased the heat transfer. This demonstrates numerically the link between fluid mixing and an increased value of heat transfer.

Practically speaking, the forced convection heat transfer may or may not increase with concentration of nanoparticles. In general, energy transportation between nanofluid layers would increase by increasing the concentration of nanoparticles. This could possibly be explained by several mechanisms. One such mechanism is the collisions and the random motion of nanoparticles which will transfer the energy across the nanofluid and increase the energy transportation. Simultaneously, nanofluid viscosity increases with concentration of nanoparticles and this increased viscosity may also play a role in explaining the effects of nanoparticle concentration. The detail of effects of nanoparticle concentration on nanofluid viscosity discussed in reference [8]. Fluctuation of the fluid flow and consequently the energy transportation between fluid layers would be suppressed with nanofluid viscosity. One may conclude that the reduction or enhancement of nanofluid forced convection heat transfer as a function of nanoparticle concentration may depend on which factor is dominant. The nanofluid heat transfer coefficient would decrease with nanoparticle concentration, if enhancement of nanofluid viscosity becomes dominant and nanofluid heat transfer coefficient would increase with nanoparticle concentration, if energy transfer between fluid layers becomes dominant.

### 1.2. Effects of Laminar Forced Convection Flow on Nanofluid Heat Transfer

**Figure 3:**
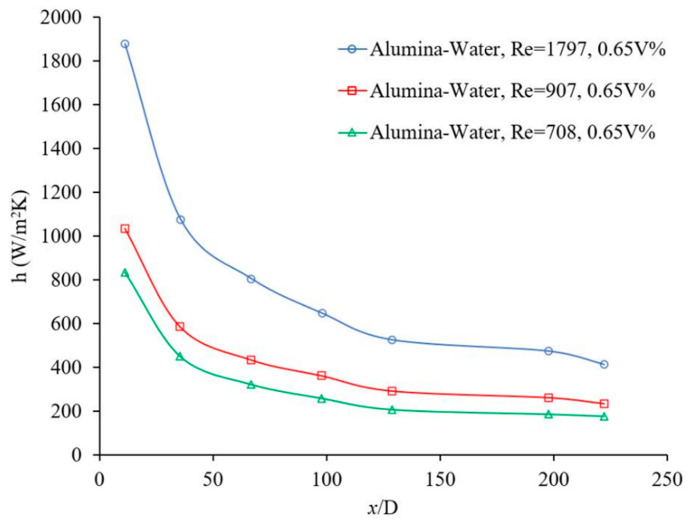
Effect of Reynolds number on heat transfer coefficient, Rea et al. [14].

**Figure 4:**
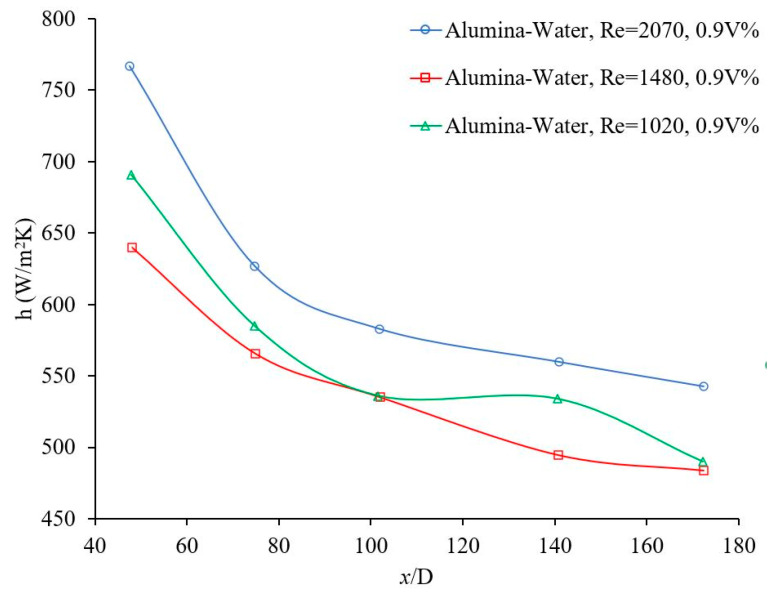
Effect of Reynolds number on heat transfer coefficient, Noghrehabadi and Pourrajab [15].

**Figure 5:**
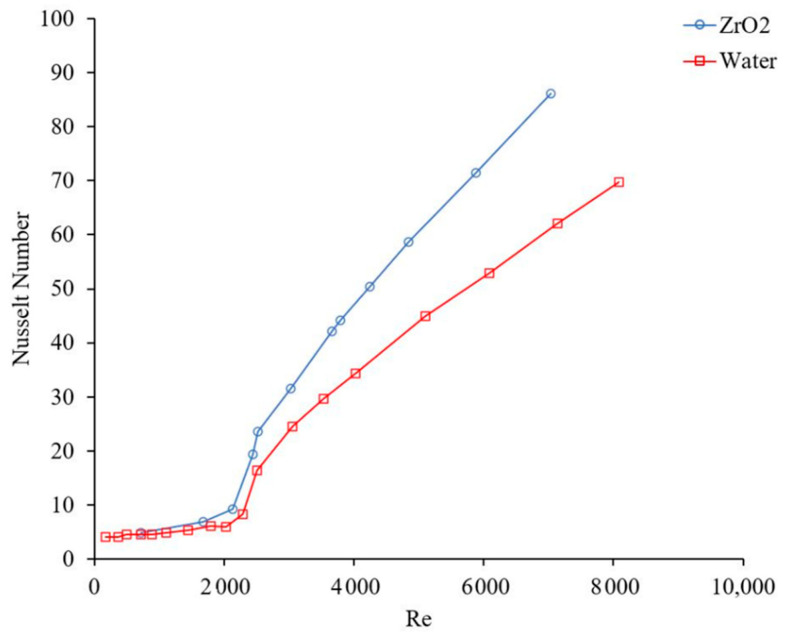
Effect of Reynolds number on heat transfer coefficient, mass concentration 9 wt %, Haghighi et al. [28].

**Figure 6:**
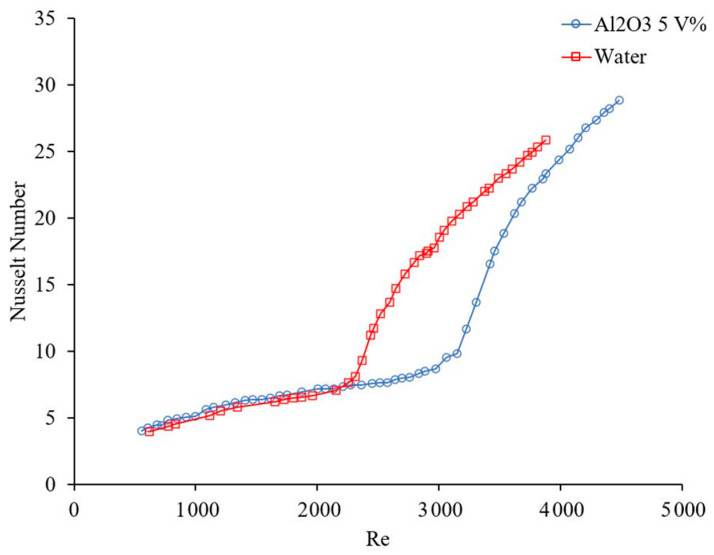
Effect of Reynolds number on heat transfer coefficient, Liu and Yu [17].

The flow regime has an important role on random motions of particles and consequently on the nanofluid heat transfer coefficient. Figure 3 and Figure 4 show the effects of Reynolds number, which indicates flow regime, on the forced convection heat transfer coefficient. In general, the heat transfer coefficient increases with Reynolds number in laminar and turbulent flows, since energy transfer as a result of random flow motion increases compared to viscous effects. Similar results were observed by many researchers [4,17,18,19]. Figure 5 and Figure 6 shows the variation of the Nusselt number as a function of distance in transient flow condition. These figures clearly explain that there is a need to study the effects of combinations of parameters, instead of the effects of one parameter at a time. It was reported that adding nanoparticles may enhance [28] or deteriorate [17] the heat transfer coefficient in transient flow conditions. If the effect of random flow motion is dominant in energy transportation, the nanofluid heat transfer coefficient may increase in transient flow condition otherwise the heat transfer coefficient may deteriorate by introducing nanoparticles in the base liquid. Adding nanoparticles may have negative side effects by enhancing the nanofluid viscosity and consequently, suppress the random flow motion or random motion of nanoparticles inside the base liquid.

The convective heat transfer coefficient of alumina-water nanofluid was investigated in rectangular microchannels at a laminar flow regime when, 5<Re<300, where the average alumina nanoparticle size was ~170 nm [20]. The local heat transfer coefficient as a function of axial distance for different nanoparticle volume fractions and different Reynolds numbers was measured inside (a) 50 × 50 $\mu m^{2}$, Re = ~14.8 and ~83.3, base liquid was water (b) 100 × 100 $\mu m^{2}$, Re = ~59.9 and ~286.6, base liquid was water and (c) 100 × 100 $\mu m^{2}$ for Re = ~6 and ~32, the base liquid was a mixture of 50% water and 50% ethylene glycol. The heat transfer coefficient increased with nanoparticle volume fraction and Reynolds number; and the effect of the entrance region was stronger at higher Reynolds numbers. No entrance region effect was observed for (a) Re = ~6, (b) Re = ~14.8, and (c) Re = ~59.9. Perhaps, the ethylene glycol was not a suitable base fluid in current conditions because of lack of fluid flow stability. The average heat transfer coefficient as a function of Reynolds number was measured for different nanoparticle volume fractions and different channel sizes. The nanofluid heat transfer coefficient was higher than that of the base liquids in all cases. For a given Reynolds number, the average heat transfer coefficient increased as channel size decreased. The slope of variation of the average heat transfer with Reynolds number decreased as channel size increased [20]. Another investigation was conducted to study the laminar convective heat transfer of TiO_2_–water nanofluid inside a 7.8 mm uniformly heated tube experimentally and numerically. The average TiO_2_ nanoparticle size was 21 nm [4]. It was investigated (a) the variation of local heat transfer coefficient with axial distance from entrance for different Reynolds numbers and nanoparticle volume fractions (1 vol %, 1.6 vol %, and 2.3 vol %) and (b) the variation of average heat transfer coefficient with Reynolds number for different nanoparticle volume fractions. It was found that (a) the local heat transfer increased with Reynolds number and nanoparticle volume fraction and (b) the heat transfer coefficient enhancement was more noticeable at the entrance region. Plotting the average heat transfer coefficient as a function of Reynolds number for different nanoparticle volume fractions, indicated similar results with clear observation of nanoparticles effects. The maximum average heat transfer coefficient enhancement was 21%, at a nanoparticle volume fraction of 2.3 vol %, where Reynolds number was ~940. The average heat transfer coefficient was enhanced with the nanoparticle volume fraction for all cases consistently [4]. It was indicated that thermal conductivity enhancement was not the only responsible reason for the nanofluid heat transfer coefficient enhancement. Different possible mechanisms for the remarkable nanofluid heat transfer enhancement were proposed including thermal conductivity enhancement, Brownian motion of the nanoparticles, the non-uniform shear rate of nanofluids which leads to the reduction of viscosity in the vicinity of the tube wall, and migration of nanoparticles. In case of the numerical investigations, the single-phase model was compared with the common two-phase numerical approaches. The predicted heat transfer coefficients, using single-phase and common two-phase approaches, underestimated and overestimated the experimental data, respectively. The two-phase model was modified to enhance the level of accuracy for the prediction of the nanofluid heat transfer. The modified model predicted that the convective heat transfer coefficient increased with nanoparticle concentration and Reynolds number, and decreased with increasing nanoparticle size. Therefore, nanofluids with smaller nanoparticles size and higher thermal conductivity were recommended for solar liquid heating collectors [4]. In the same way, an experiment was conducted to investigate the variation of the local heat transfer coefficient as a function of axial distance and the average heat transfer coefficient as a function of Reynolds number for different nanoparticle volume fractions [29]. 20 nm Cu_2_O nanoparticles were mixed with deionized water to produce nanofluid with volume fractions of 1 vol %, 2 vol %, and 4 vol %. The axial variation of the measured nanofluid local heat transfer coefficient was presented for various volume fractions and Reynolds numbers in the laminar flow regime. It was explained that (a) the nanofluid heat transfer coefficient increased with nanofluid thermal conductivity enhancement and decreased with the increase of the thermal boundary layer thickness; (b) the effect of the nanofluid thermal conductivity enhancement was dominant for the given conditions, therefore the nanofluid heat transfer coefficient was enhanced with nanoparticle volume fraction. The local heat transfer coefficient was measured for different Reynolds numbers and volume fractions, at x/D=8.8. The maximum heat transfer coefficient enhancement was 61% where the particle volume fraction was 4% and Reynolds number was ~605. It was observed that the heat transfer coefficient increased with the Reynolds number and volume fraction for all cases consistently. Similarly, the average heat transfer coefficient as a function of Reynolds number was measured for different nanoparticle volume fractions. It was found that (a) the average heat transfer coefficient increased with the volume fraction for a given Reynolds number and (b) the average heat transfer coefficient increased with Reynolds number for a given particle volume fraction [29]. Similarly, the local alumina–water nanofluid heat transfer coefficient against the axial distance was measured for different nanoparticle volume fractions inside a 4.5 mm pipe at Re=1050 and 1600. The alumina nanoparticle size was in range of 27–56 nm [30]. In addition, the local heat transfer coefficient as a function of axial distance was measured for different nanoparticle volume fractions. The results indicated that: (a) The thermal developing length of nanofluids was greater than that of the base liquid and the thermal developing length of nanofluid increased with an increase of particle concentration. (b) The nanofluid enhanced the heat transfer coefficient significantly, particularly at the entrance region and at higher Reynolds numbers. The nanofluid local heat transfer coefficient enhancement, at x=285 mm and φ=1.6%, was 41% and 47% at Reynolds numbers 1050 and 1600, respectively. (c) The local heat transfer coefficient increased with the nanoparticle volume fraction consistently. (d) The local heat transfer coefficient enhancement was much greater than the thermal conductivity enhancement for given conditions. (e) The local heat transfer coefficient decreased with axial distance for all cases. The nanofluid local heat transfer coefficient enhancement at Re = 1600 and φ=1.6%, was 47% and 14% at x=285 mm and ~778.5 mm, respectively. Also, the Nusselt number was measured against the Reynolds number for different nanoparticle volume fractions at laminar flow. It was observed the nanofluid Nusselt number was higher than that of water; and the Shah correlation failed to predict the nanofluid Nusselt number. It was proposed that the nanofluid heat transfer coefficient enhancement was because of nanofluid thermal conductivity enhancement as well as particle migration [30]. Furthermore, the convective heat transfer coefficient of graphene–water nanofluid in a laminar flow through a circular pipe (4.2 mm internal diameter) with uniform wall heat flux was investigated experimentally [16]. The local Nusselt number as a function of axial distance was measured for distilled water and results were compared with the prediction of the Shah correlation [23,31] to evaluate the accuracy and reliability of the experimental setup. The maximum relative error between the measured and predicted Nusselt numbers was about 6%. Variation of the local heat transfer coefficient with axial distance for different nanoparticle volume fractions was investigated at Re = 1025 and it was found that adding small amount (up to 0.02% volume fraction) of Graphene nanoparticles into the water increased the local heat transfer coefficient dramatically. The local heat transfer enhancement was observed to be more noticeable in the entrance region. For instance, at x/D=12.9, the local heat transfer enhancements were 17.9%, 22.5%, and 26.0% for nanoparticle volume fraction of 0.005 vol %, 0.01 vol %, and 0.02 vol % respectively. The local heat transfer enhancements for x/D=380.3 were decreased to 5.2%, 8.7%, and 11.6%, respectively. It was suggested that the heat transfer coefficient enhancement with increase in nanoparticle volume fraction was due to the nanofluid thermal conductivity enhancement and the reduction of thermal boundary layer thickness. Also, the Nusselt number as a function of Reynolds number was plotted for different nanoparticle volume fractions. The Nusselt number increased with Reynolds number for a given nanoparticle volume fraction. However, the nanoparticle volume fraction did not have a consistent effect on the variation of the Nusselt number against the Reynolds number. It was found that the impact of nanoparticles below and above Re = 1200 were different. Below Re = 1200, the water Nusselt number was higher than the nanofluid Nusselt number and vice versa above Re = 1200. The variation of the average nanofluid heat transfer coefficient as a function of Reynolds number for different nanoparticle volume fractions showed the effects of Reynolds number and nanoparticle volume fraction clearly. The maximum nanofluid thermal conductivity enhancement was 10.3% at 0.02 vol % and the maximum nanofluid heat transfer coefficient enhancement was 14.2% at 0.02 vol % where Reynolds number was 1850 [16]. In a similar way, laminar flow experiments in a uniformly heated pipe (4 mm internal diameter) were carried out to investigate the effects of 30 nm CuO nanoparticle volume fraction and Reynolds number on the local and average heat transfer coefficients [32]. To validate the accuracy and reliability of the experimental setup, the local heat transfer coefficient as a function of axial distance for pure water at Re = 1700 was measured and compared with a semi-empirical correlation [33]. A relative agreement between the experimental data and the prediction was observed and maximum deviation was ~6%. Also, the Nusselt number as a function of Reynolds number (350 to 2000) for pure water was measured and compared with a semi-empirical correlation [34]. A relative agreement between the experimental data and the prediction was observed and the maximum deviation was ~5%. The local heat transfer coefficient was measured as a function of axial distance in the entrance region for pure water and 2 vol % CuO-water nanofluid with two mass flow rates (104 gr/min and 194 gr/min). The local heat transfer coefficient increased with the nanoparticle volume fraction and the mass flow rate in the entire entrance region. 2 vol % CuO-water nanofluid enhanced the local heat transfer about 12% along the entire channel with respect to pure water. In addition, the average heat transfer coefficient was measured as a function of mass flow rate and Reynolds number. The average heat transfer coefficient increased with the mass flow rate. The experimental results indicated that (a) for 2 vol % nanoparticle volume fraction and a given mass flow rate the heat transfer coefficient was about 13% higher than that of water, (b) for 2 vol % nanoparticle volume fraction and a given Reynolds number the heat transfer coefficient was more than 40% higher than that of water and (c) for 0.25 vol % nanoparticle volume fraction the laminar-turbulent transition was slightly delayed. For pure water, the laminar-turbulent transition occurred when the Reynolds number was over 2000 as it was expected [32].

To investigate the effects of the turbulator a twisted tape was inserted into the heating pipe in size of 7.0035 mm. The purpose of the turbulator is to twist the flow and generate turbulence inside the pipe at a low Reynolds number. The effects of the thickness of the twisted tape were investigated on the local and average nanofluid heat transfer coefficients. To validate the reliability and accuracy of experimental setup, the Nusselt number as a function of axial distance from entrance was measured for pure water inside the plain tube, at Re=919.3, and compared with prediction of Shah correlation [23]. A good agreement was observed between the experimental data and the prediction. The deviation of the experimental Nusselt number and the Shah correlation was in range of 0.1–5.1%. The average heat transfer coefficient was measured against the Reynolds number, for 0.1 vol % nanoparticle volume fraction with and without twisted tape, and with different twisted tape thicknesses (0.5 mm, 1 mm, and 2 mm). The average heat transfer coefficient increased with Reynolds number and the thickness of the twisted tape. The plain tube had the lowest average heat transfer coefficient for all Reynolds numbers. For a given tube and mass flow rate, as the thickness of the twisted tape increased, the cross-sectional area of the fluid decreased, the velocity profile changed, and the average velocity increased which led to better cross mixing in the vicinity of tube wall and consequently enhanced the average heat transfer coefficient. Also, the local heat transfer coefficient was measured as a function of axial distance for different twisted tape thicknesses and for different working fluids such as water and alumina nanofluids (0.5 vol % and 1 vol %). The average alumina nanoparticle size was 15 nm. It was found that (a) the heat transfer coefficient increased with the thickness of the inserted twisted tape for a given nanoparticle volume fraction. The experimental results indicated that: (a) the pure water heat transfer coefficient enhancements were 75.03%, 80.20%, and 90.58% respectively for twisted tape thickness of 0.5 mm, 1 mm, and 2 mm; (b) for a given thickness of twisted tape, the heat transfer coefficient increased with nanoparticle volume fraction; and (c) the maximum heat transfer coefficient enhancement occurred in the entrance region and then heat transfer coefficient enhancement reduced with axial distance from pipe entrance [35].

### 1.3. Effects of Nanoparticle Size and Material

**Figure 7:**
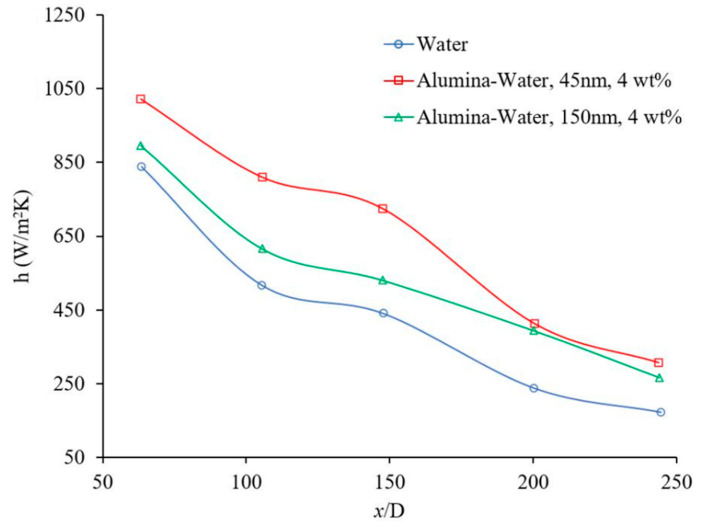
Effect of nanoparticle size on heat transfer coefficient, Anoop et al. [37].

Nanoparticle size has a significant effect on nanofluid physical properties and the nanofluid forced convection heat transfer. Figure 7 shows the effects of nanoparticle size on the heat transfer coefficient. There have been cases where it was observed that the heat transfer coefficient increases with nanoparticle size and as nanoparticle concentration increases the effects of nanoparticle size increases [36]. However, in this figure, it was observed that as nanoparticle size decreases the heat transfer coefficient increases [37]. In general, nanoparticle size would change the random motion of nanoparticles, homogeneity of nanofluids and possible collision of nanoparticles, physical properties such as viscosity, and thermal conductivity. Both physical properties have significant impacts on forced convection heat transfer. Thus, the effect of nanoparticle size on the forced convection heat transfer may depend on which factor predominates under the current fluid conditions. It was observed that bigger nanoparticle size may enhance or deteriorate both viscosity and thermal conductivity of nanofluid [38,39]. In addition, as nanoparticle size increases the uniformity of the nanofluid decreases and the possibility of nanoparticle deposition increases.

Another experiment was conducted to study the effects of alumina nanoparticle size on the laminar nanofluid heat transfer coefficient [37]. The heat transfer coefficient as a function of Reynolds number (700–2100) was measured for different mass concentrations, at x/D=147. It was found that the heat transfer coefficient increased with Reynolds number and mass concentration for all cases for nanoparticle sizes of 45 nm and 150 nm. The heat transfer coefficient enhancement was higher for nanofluids with smaller nanoparticle sizes. Likewise, the local heat transfer coefficient as a function of axial distance was measured for two different particle sizes (45 nm and 150 nm). The local heat transfer coefficient increased as particle size decreased, and the heat transfer coefficient enhancement was significant in the entrance region where x/D was lower. Likewise, the average heat transfer coefficient and Nusselt number were measured as a function of Reynolds number (800–2000) at x/D=147 for different working fluids such as water, alumina nanofluids with 4 wt % nanoparticle concentration and two different nanoparticle sizes, 45 nm and 150 nm. It was indicated for x/D=147, Re=1550 and 45 nm nanoparticle size, the nanofluid heat transfer coefficient enhancement was ~25% whereas the thermal conductivity enhancement for the same conditions was ~6%. Likewise, for the 150 nm nanoparticle size, the nanofluid heat transfer coefficient enhancement was ~11% whereas the thermal conductivity increased ~4%, for similar conditions. As nanoparticle size decreased, the nanofluid thermal conductivity and nanofluid heat transfer coefficient increased. However, it was speculated that the thermal conductivity enhancement may not be the only reason for the heat transfer coefficient enhancement and perhaps particle migration and/or thermal dispersion could play an effective role as well. To investigate the effects of axial distance, the heat transfer coefficient was measured as a function of Reynolds number for three different x/D (63, 147, and 244) and for different working fluids such as water and 4 wt % alumina nanofluid where the particle size was 45 nm. At lower x/D, the heat transfer coefficient was a function of Reynolds number but at x/D>200, the heat transfer coefficient was independent of Reynolds number. The effect of nanoparticles on heat transfer coefficient enhancement was more significant in the entrance region. For instance, at Re = 1550, the heat transfer coefficient enhancement was 31%, 25%, and 10% at x/D=63, 147, and 244, respectively [37].

**Figure 8:**
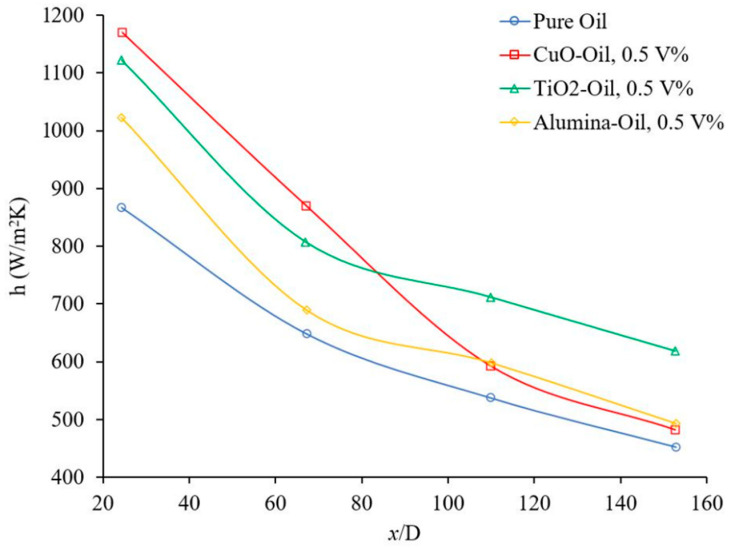
Effect of nanoparticle material on heat transfer coefficient at Re ≈ 750, Heris et al. [3].

Figure 8 shows another experiment that demonstrates the effect of nanoparticle material on the heat transfer coefficient. It was observed that nanoparticle material can play a significant role in determining the heat transfer coefficient for given conditions. Figure 8 shows that the behavior of Cu-oil nanofluid is slightly different for certain conditions which may be related to the interaction between nanoparticles and oil molecules. Nanoparticle material has a significant impact on the viscosity and the thermal conductivity of nanofluid. Both physical properties may increase or decrease the forced convection heat transfer coefficient of nanofluid, which depends on nanofluid conditions. Apparently, the effects of material nanoparticles depend on many factors including nanoparticle size which required further investigation.

### 1.4. Effects of Nanoparticle Shape

An experimental setup was built to investigate the effects of titanate nanotubes on the local heat transfer coefficient inside a pipe with a size of 3.97 mm. The aspect ratio of the titanate nanotube was ~10 (10 nm diameter and 100 nm length) [20]. The local heat transfer coefficient as a function of distance for different nanoparticle volume fractions was measured in the laminar flow regime, 1100<Re<2300. An excellent heat transfer coefficient enhancement was observed despite the small thermal conduction enhancement. For instance, the local nanofluid heat transfer enhancement at x/D=50.4 was 11.8%, 23.5%, and 24.9% at nanoparticle volume fraction of 0.12 vol %, 0.24 vol %, and 0.6 vol %, respectively. For the same conditions at x/D=453.6, the local nanofluid heat transfer enhancements were 5.6%, 13.2%, and 13.5%, respectively. Apparently, the local nanofluid heat transfer enhancement was higher at the entrance region. Also, the local heat transfer coefficient as a function of distance was measured for different Reynolds numbers. It was observed the heat transfer coefficient increased with Reynolds number for all cases consistently. Furthermore, for similar conditions, the enhancement of both the thermal conductivity and the convective heat transfer coefficient of the Titanate nanotube nanofluids were considerably higher than those of spherical Titanate nanoparticles [40] which indicates the important role of particle shape in heat transfer enhancement [20]. Likewise, the effects of multi-walled carbon nanotubes were investigated on the convective heat transfer coefficient inside a pipe with a size of 4.5 mm [41]. It was found that sodium laurate (SL), sodium dodecyl benzene sulfonate (SDBS), and gum arabic (GA) were able to stabilize the carbon nanotubes. The distilled water was mixed with multi-walled carbon nanotubes and gum arabic to produce stable nanofluid with different mass concentrations. To measure the reliability of the experimental setup, the local heat transfer coefficient as a function of axial distance for water at two Reynolds numbers (850, 1100) was measured and compared with the Shah prediction [23]. A reasonable agreement between the experimental data and the Shah prediction was observed. The nanofluid local heat transfer as a function of axial distance was measured for different carbon nanotube (CNT) concentration at Re=800. It was found that: (a) The local heat transfer coefficient increased with nanotube concentration consistently. (b) The effect of the entrance region was not very strong. (c) The heat transfer coefficient enhancement with respect to pure water increased with x/D initially, reached a maximum, and then decreased with a further increase in x/D. The axial location of maximum enhancement moved to the right side (*x* increased) as nanotube concentration increased. (d) The local heat transfer coefficient increased with Reynolds number, 1000<Re<1200. The variation of the local heat transfer coefficient was more complex at Re=800. (e) The maximum heat transfer coefficient enhancement was over 350% at ~x/D=110, where Re=800 and CNT concentration was 0.5 wt %. It was suggested that the heat transfer coefficient enhancement could not be attributed only to the thermal conductivity enhancement under static conditions. Particle rearrangement, shear induced thermal conduction enhancement, the reduction of thermal boundary layer thickness, and the high aspect ratio of nanotube were proposed to be possible mechanisms of the heat transfer coefficient enhancement [18].

A summary of the various experimental studies that are presented in this literature review which deals with heat transfer coefficient can be found in Table A1 in the Appendix A. This table presents the essential components of the experimental set up. It also provides a brief summary of the main points of the study.

## 2. Materials and Methods

### 2.1. Nanofluid Preparation and Related Calculations

**Figure 9:**
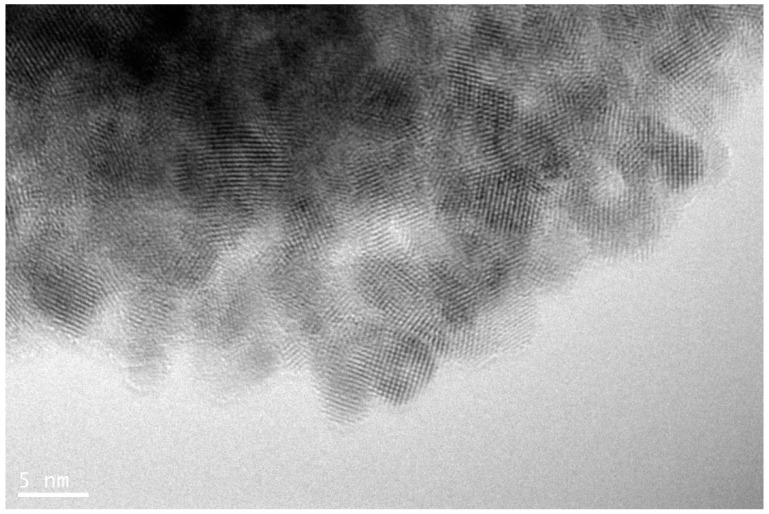
TEM image of TiO_2_ nanoparticles.

The nanofluids used in this experiment were created in two ways. All Al_2_O_3_ nanoparticles and some TiO_2_ particles were produced by mixing a base liquid with the premade nanoparticles. The base liquid and the nanoparticles were carefully measured using an electric balance and then nanoparticles were mixed with base liquid, using a magnetic stirrer. The anatase TiO_2_ which were man-made by combining reagent grade TiCl_4_ (solution, ~16% Ti from Wako Chemicals, Richmond, WV, USA), methanol (Fisher, Hampton, NH, USA), and sodium hydroxide NaOH (Sigma-Aldrich, St. Louis, MO, USA) without further purification to make the solution. First, 0.465 M aqueous NaOH solution was mixed with methanol in a 2:5 volume ratio. 57.5 g of the TiCl_4_ solution was then mixed dropwise into the NaOH/deionized water/methanol mixture under ambient conditions during stirring. Then deionized water was added until a final volume of 500 mL was reached. The mixture was stirred for three days at a constant temperature of 35 °C. A centrifuge was used to separate the semiconductor TiO_2_ nanoparticles from the base liquid. The separated nanoparticles were mixed with deionized water and separated again, using centrifuge. Eventually the TiO_2_ nanoparticles were dried, using the dry-freezing apparatus. Nanoparticles were not coated and it was observed that the nanoparticles distributed uniformly in most of base liquids, including water. The crystal phase of TiO_2_ nanoparticles found using XRD method which is explained in reference [42] in detail. Figure 9 shows TEM image of TiO_2_ nanoparticles. The nanoparticles are almost spherical in size of ~5–10 nm. The detail of this nanoparticle production can be seen in reference [42].

The man-made rutile TiO_2_ nanorods produced by mixing 0.465 M aqueous NaOH solution with methanol in a 2:5 volume ratio. Under ambient conditions and constant stirring 57.5 g of the TiCl_4_ solution added dropwise to the NaOH/water/methanol mixture. After mixing was completed, deionized water was added so that the final volume of the reaction mixture was 500 mL. The final reaction mixture heated for 7 days at 27 °C. Similarly, the base liquid separated from rutile TiO_2_ nanorods, using centrifuge and eventually dried by evaporation [43,44]. The rutile TiO_2_ nanorods have ~20 nm length and ~5 nm diameter.

Before each test, the nanofluid was remixed using an ultrasonic bath to ensure homogeneity and combat any possible precipitation. All used nanoparticles were stable in the base liquid and no sedimentation observed for 24 h. The syringe used in the syringe pump was also cleaned before each test. Deionized water was loaded into the syringe and run through the microchannel assembly to clean off any deposition within the microchannel and plastic tubing. Once the assembly was cleaned nanofluid was loaded into the syringe and run through the system. For each test, the flow rate was initially started at a low value where there would be a low Reynolds number. This flow rate was then gradually increased until it reached the desired flow rate for the test. After every run, the entire system was flushed using deionized water. The syringe was filled with deionized water which was run through the system at high flow rates for up to 15 min to minimize the deposition of nanoparticles on the inner walls of the microchannel.

Density and specific heat of nanofluids can be calculated using Equations (1) and (2), respectively [14].

(1) $$\rho_{nf}=\phi \rho_{p}+\left(1-\phi \right)\rho_{bf}$$

(2) $$c_{p_{nf}}=\frac{\phi {\left(\rho c_{p}\right)}_{p}+\left(1-\phi \right){\left(\rho c_{p}\right)}_{bf}}{\rho_{nf}}$$

(3) $$\phi =\frac{w\rho_{bf}}{\rho_{p}\left(1-w\right)+w\rho_{bf}}$$

In general, the viscosity of nanofluids were calculated, using well-known correlations [45] or found from literature [46,47] for given conditions. The viscosity of ethanol-TiO_2_ [44], ethanol-Al_2_O_3_ [44], water-TiO_2_ [45], and water-Al_2_O_3_ [46] were found from given references. The thermal conductivity of ethanol-TiO_2_, ethanol-Al_2_O_3_, water-TiO_2_, and water-Al_2_O_3_ were found from reference [44]. Table 1 and Table 2 would provide the characteristics of nanoparticle and physical properties of used nanofluids in this study. In the case of commercial nanoparticles, the specifications are obtained from the producer.

The inlet and outlet temperatures were measured, using thermocouples. Using inlet and outlet temperatures, mass flow rate of the fluid, and specific heat of the nanofluid, the rate of energy absorption and heat flux can be calculated using Equations (4) and (5), respectively. Physical properties of nanofluids provided in Table 2.

(4) $${\dot{Q}}_{absorbed}=\dot{m}c_{p}\Delta T=\dot{m}c_{p}\left(T_{out}-T_{in}\right)$$

(5) $$\dot{q}={\dot{Q}}_{absorbed}/A$$

$\dot{q}$ is heat flux and A is the heat transfer area. Knowing the inlet temperature, the fluid temperature as a function of distance within the pipe can be determined using Equation (6).

(6) $$T\left(x\right)=T_{in}+\frac{\dot{q}\pi D}{c_{p}\dot{m}}x$$

Distribution of the surface temperature as a function of distance was measured, by attaching thermocouples on surface of microchannel. Knowing the distribution of the temperature along the microchannel, the heat transfer coefficient can be calculated from Equation (7).

(7) $$h\left(x\right)=\frac{\dot{q}}{\left(T_{s}\left(x\right)-T_{f}\left(x\right)\right)}$$

The measured temperatures were recorded, when fluid flow was in steady state condition. For a given condition, the heat transfer coefficient as a function of location is calculated. The standard deviation of calculated heat transfer coefficients was calculated and it was observed that the standard deviation of heat transfer coefficient for a given point is small relative to value of heat transfer coefficient for the given point. Therefore, the error bars were ignored.

### 2.2. Experimental Setup

**Figure 10:**
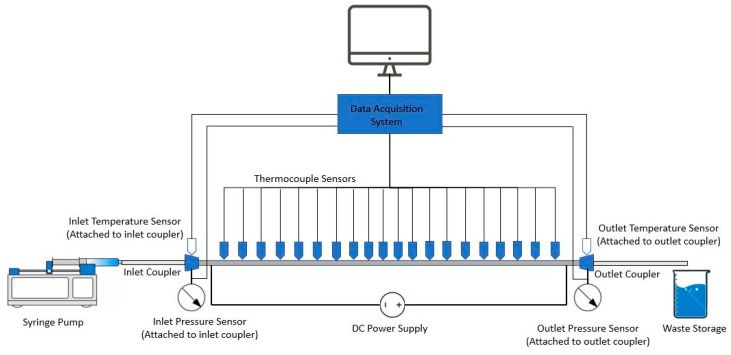
Experimental setup diagram.

Figure 10 shows the experimental set up which was used to determine the forced convection heat transfer coefficient. Fluid was pumped from a New Era Pump Systems’ NE-1000 syringe pump, with a 100 mL Hamilton syringe. The NE-1000 pump has a range of flow rates from 40 µL/h to 2900 mL/h. The working fluid was pumped through a section of plastic tubing (Hamilton 86510, De Pere, Wisconsin, USA) into a four-way junction (Upchurch Scientific 5700184, Stockbridge, Georgia, USA) where temperature and pressure sensors monitored inlet fluid conditions. The pressure sensor and the inlet thermocouple each occupied one of the four channels of the inlet junction. The other two were occupied by the inlet tube and the microchannel. The distribution of temperature along the microchannel was measured by attaching 86 μm thermocouples on topside of channel, using high thermal conductive glue. The distance between thermocouples was 1 cm.

The pressure sensor used was an Omega PX26-100GV pressure transducer (Norwalk, CT, USA) powered by an Omega PST 4130 power supply (Norwalk, CT, USA). This power supply was tuned to an output of 12 volts DC and 150 mA. The temperature sensor used was an Omega 5TC-TT-K-30-36 0.2 µm thermocouple (Norwalk, CT, USA). After passing through inlet junction the fluid entered the microchannel body. The microchannel used in this experiment is a 25-cm-long stainless-steel needle which is oriented horizontally as shown in Figure 10. The inner diameter of the channel is 210 μm. The 304 stainless-steel needles purchased from Hamilton Company (De Pere, WI, USA). Like the inlet, pressure and temperature sensors were used at the four-way outlet junction (Upchurch Scientific 5700184, (Stockbridge, GA, USA)) in order to determine the outlet pressure and outlet temperature. Once the fluid passed the outlet junction more plastic tubing fed the fluid into a waste storage beaker for disposal. The data acquired from the temperature and pressure sensors at the inlet and outlet was used in conjunction with the set flow rate to determine the rate of power absorption by the working fluid. At the inlet and outlet junctions the high pressure drops experienced due to the small cross section of the microchannel could cause leakage at the temperature and pressure inputs of the junction. This is counteracted by using Loc-Tite epoxy to ensure the flow remains in the desired path.

#### 2.2.1. Heating Elements and Sensors

Along the microchannel 20 RS Pro 397-1589 86-μm diameter thermocouples were attached using high thermal conductivity glue. The thermocouples were positioned on top of the channel, along the highest point, and were situated equidistant from each other at intervals of 1 cm. Each thermocouple was connected to the surface of the channel with high thermally conductive Cotronics Duralco 132 epoxy (Brooklyn, NY, USA). The epoxy ensured the thermocouples would maintain contact with the surface while minimizing heat loss. A layer of 3M scotch-weld 2214 epoxy glue and dry insulation was added to the channel to further minimize the effect of ambient conditions. The data from these thermocouples was used to generate temperature profiles along the length of the pipe.

The test section of the microchannel was heated resistively using a Sorensen XPH 20-20 DC power supply (Ameteck, Berwyn, PA, USA). The power supply was rated for volt loads of 0–20 V and amperages of 0–20 A. The voltage and amperage values are shown on the display of the power supply. The wires of the power supply were attached directly to the to the stainless steel microchannel. The voltage supplied by the power supply caused the microchannel to heat up, which in turn heated the fluid in the channel. The values of voltage and current were used to calculate the generated heat through the law of conservation of energy. The resistance across the pipe was measured using a National Instruments USB-4065 Digital Multimeter (Austin, TX, USA) attached between the power supply wires and the thermocouples, with the positive and negative terminals attached correspondingly to the power supply wires across the channel to measure resistance. During a test, the power supply was initially set at zero. The power was then gradually increased until the desired heat flux was achieved. The fluid was carefully monitored to ensure its temperature remained below 90 °C. 

#### 2.2.2. Data Acquisition Instrumentation

The thermocouple sensors along the channel are connected to a LabVIEW 2012 logging software (Austin, TX, USA) through a National Instruments NI 9213 card and a National Instruments NI cDAQ-9178 base. The power transducers are fed into the same base using the NI 9218 with NI9982 adapters. The voltage drop across the entirety of the microchannel is measured using a National Instruments NI 9221 card. Once the system reached a steady state of operation during a test, the data acquisition system was activated and data was recorded. Data is logged using the LabView system then saved as a spreadsheet for post analysis.

## 3. Results and Discussion

**Figure 11:**
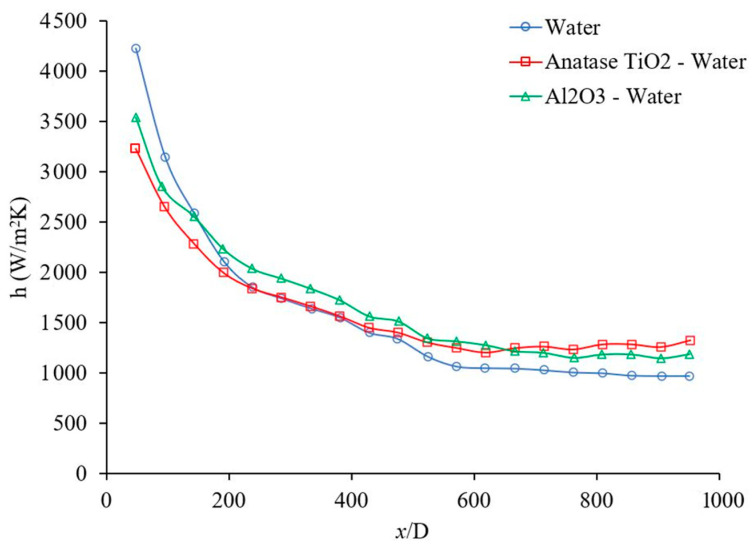
Variation of heat transfer coefficient as a function of distance for Al_2_O_3_–water and TiO_2_–water nanofluids. Nanofluid concentration was 1 wt %, and the range of Reynolds number inside the microchannel was 150–200. The Al_2_O_3_ and Anatase TiO_2_ nanoparticles were commercial.

**Figure 12:**
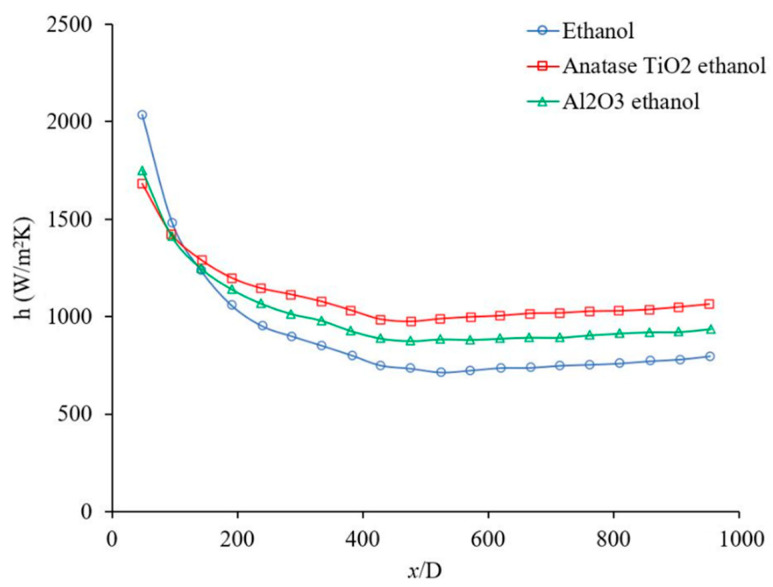
Variation of the heat transfer coefficient as a function of distance for Al_2_O_3_–ethanol and TiO_2_–ethanol nanofluids. Nanofluid concentration was 1 wt %, and the range of Reynolds number inside the microchannel was 150–200. The Al_2_O_3_ and Anatase TiO_2_ nanoparticles were commercial.

The forced convection heat transfer coefficient was measured as a function of distance for different base liquids and nanofluids. The concentration of the nanofluids were 1 wt % and the range of Reynolds number inside the microchannel was 150–200. Figure 11 indicates the heat transfer coefficient as a function of distance for Al_2_O_3_–water and TiO_2_–water nanofluids. Figure 11 demonstrates that Al_2_O_3_ and TiO_2_ nanoparticles enhance the heat transfer coefficient in the fully developed region where the profile of velocity remains constant as *x* increases. As it can be seen in Figure 11, Al_2_O_3_ and TiO_2_ nanoparticles enhanced the heat transfer coefficient in the fully developed region by 21%, and 33%, respectively when base liquid was water. As fluid moves forward the fluid temperature increases and as results the viscosity of nanofluid decreases which reduces the suppression of random motion of nanoparticle, nanoparticle–nanoparticle, and nanoparticle–molecule interactions. Therefore, the nanofluid heat transfer coefficient increases, compared to pure base liquid in a fully developed region. A similar phenomenon can be observed in Figure 12.

Figure 12 shows the heat transfer coefficient as a function of distance for Al_2_O_3_–ethanol and TiO_2_–ethanol nanofluids in developing and developed regions. Figure 12 demonstrates that Al_2_O_3_ and TiO_2_ nanoparticles enhance the heat transfer coefficient. The figure shows that Al_2_O_3_ nanoparticles enhanced the heat transfer coefficient in the developing region 8%, 13%, and 16%, when x/D was 191, 285, and 381, respectively, also the heat transfer coefficient was enhanced in fully developed region by 20%. Similarly, TiO_2_ nanoparticles enhanced the heat transfer coefficient in developing region 13%, 24%, and 29% when x/D was 191, 285, and 381 respectively, also the heat transfer coefficient was enhanced in fully developed region by 36%. Comparing Figure 11 and Figure 12, one can see that Al_2_O_3_ and TiO_2_ nanoparticles enhanced the heat transfer coefficient of ethanol more than that of water. Perhaps Al_2_O_3_ and TiO_2_ nanoparticles disperse more uniformly in ethanol than in water, which might be related to the lower viscosity of ethanol based nanofluids (see Table 2).

**Figure 13:**
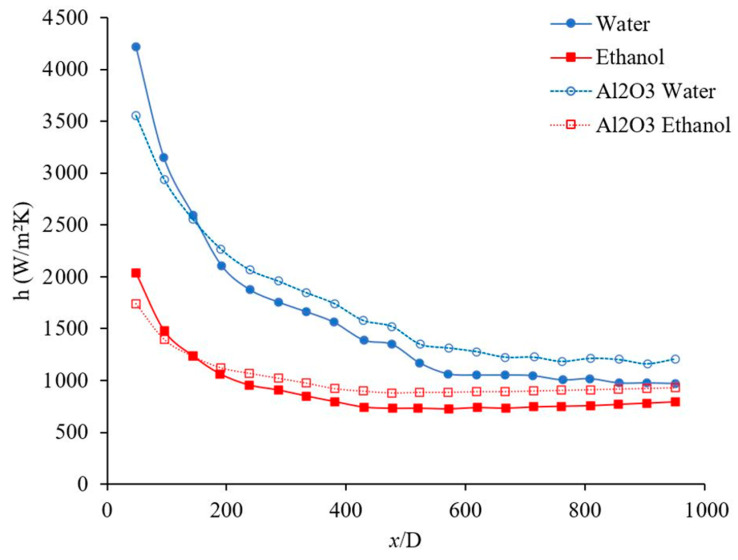
Variation of forced convection heat transfer coefficient as a function of distance for Al_2_O_3_–water and Al_2_O_3_–ethanol nanofluids. Nanofluid concentration was 1 wt %, and the range of Reynolds number inside the microchannel was 150–200. The Al_2_O_3_ nanoparticles were commercial.

**Figure 14:**
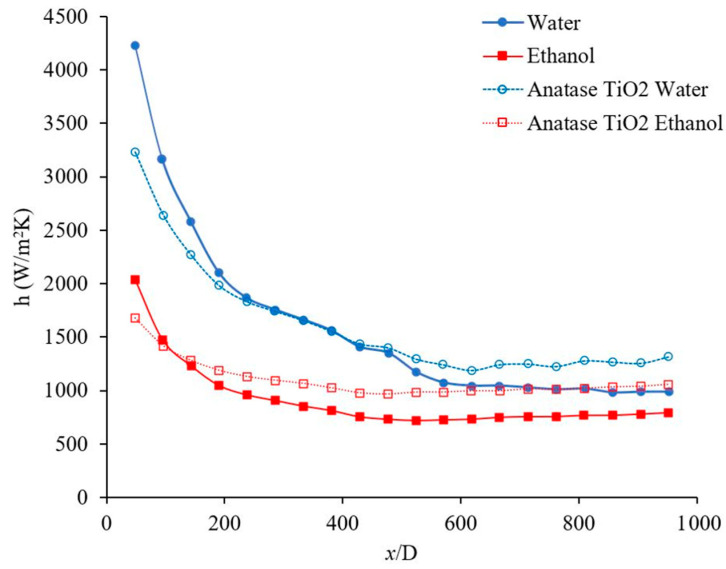
Variation of forced convection heat transfer coefficient as a function of distance for TiO_2_–water and TiO_2_–ethanol nanofluids. Nanofluid concentration was 1 wt %, and the range of Reynolds number inside the microchannel was 150–200. The Anatase TiO_2_ nanoparticles were commercial.

**Figure 15:**
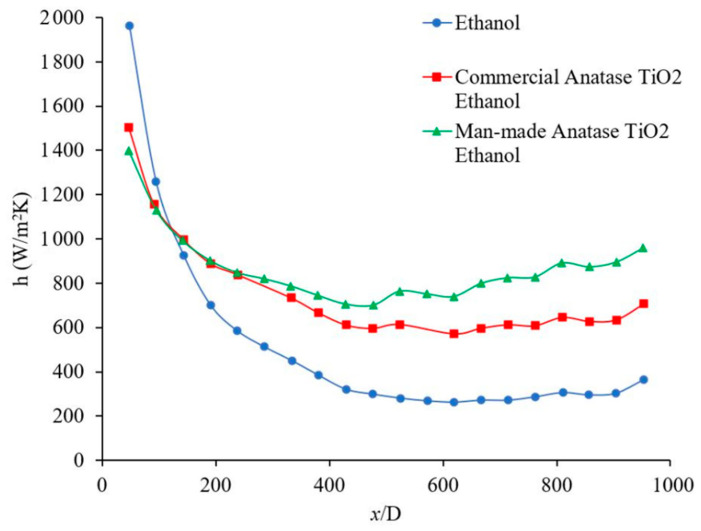
Variation of forced convection heat transfer coefficient as a function of distance for TiO_2_–ethanol nanofluids. Nanofluid concentration was 1 wt %, and the Reynolds number inside the microchannel was about 153. Comparison between effects of semiconductor anatase TiO_2_ nanoparticles made in our research group [40] and commercial anatase TiO_2_ nanoparticles.

**Figure 16:**
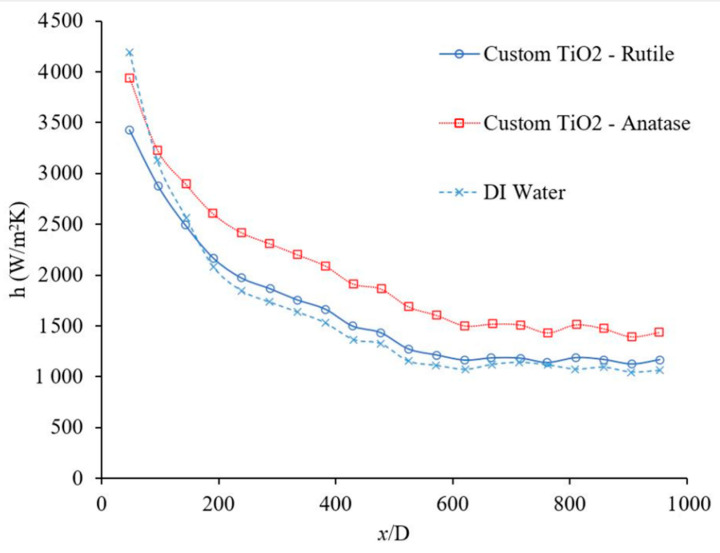
Variation of forced convection heat transfer coefficient as a function of distance for water-based nanofluids using 1 wt % nanofluid and the range of Reynolds number inside the microchannel was 150–200. Comparison is between two variations of semiconductors TiO_2_ nanoparticles made in our research group [40].

**Figure 17:**
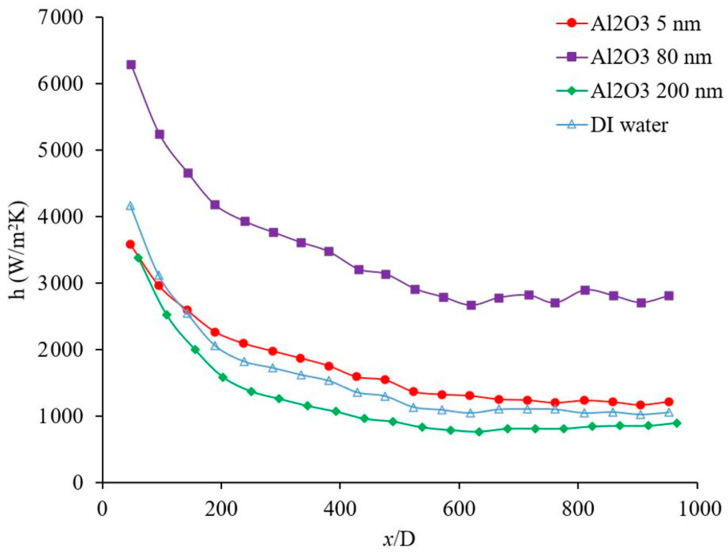
Variation of forced convection heat transfer coefficient as a function of distance for Al_2_0_3_-water nanofluids. Nanofluid concentration was 1 wt %, and the range of Reynolds number inside the microchannel was 150–200. Comparison between effects of Al_2_O_3_ nanoparticles of sizes varying from 5 nm to 200 nm.

Figure 13 shows the effects of the base liquid on the heat transfer coefficient as a function of distance. For the given conditions, it was observed that the heat transfer coefficient was higher when water was used as the base liquid, which may be related to its higher thermal conductivity. The thermal conductivity of the base liquid plays a significant role on the forced convection heat transfer coefficient. In this figure, it was observed that the heat transfer coefficient enhanced in developing region 106%, 100%, and 93%, when x/D was 48, 191, and 334, respectively, also heat transfer coefficient was enhanced in fully developed region by 26% when water used as a base liquid. Figure 13 shows the heat transfer coefficient as a function of distance for Al_2_O_3_–water and Al_2_O_3_–ethanol nanofluids. In this figure, it was observed that Al_2_O_3_ nanoparticles enhanced the heat transfer coefficient in developing region 10%, 11%, 14%, when x/D was 238, 334, and 429, respectively, also heat transfer coefficient was enhanced in fully developed region by 21% when water used as a base liquid. Similarly, Al_2_O_3_ nanoparticles enhanced the heat transfer coefficient in developing region 12%, 15%, 21%, when x/D was 238, 334, and 429, respectively, also heat transfer coefficient was enhanced in fully developed region by 19% when ethanol used as a base liquid. Al_2_O_3_–water nanofluid showed a higher heat transfer coefficient with respect to Al_2_O_3_–ethanol in the fully developed region by 30% and in the developing region by 93%, 89%, and 76%, when x/D was 238, 334, and 429, respectively. Figure 14 shows the forced convection heat transfer coefficient as a function of distance for TiO_2_–water and TiO_2_–ethanol nanofluids. In this figure, it was observed that TiO_2_ nanoparticles enhanced the heat transfer coefficient in developing region ~0% when x/D was 285, and 381, respectively, also heat transfer coefficient was enhanced in fully developed region by 30% when water used as a base liquid. Similarly, TiO_2_ nanoparticles enhanced the heat transfer coefficient in developing region 21% and 26% when x/D was 285 and 381, respectively, also heat transfer coefficient was enhanced in fully developed region by 34% when ethanol used as a base liquid. TiO_2_–water nanofluid showed higher heat transfer coefficient with respect to TiO_2_–ethanol in fully developed region by 23% and in developing region 59% and 52% when x/D was 285 and 381, respectively. Figure 13 and Figure 14 indicate that Al_2_O_3_ and TiO_2_ nanoparticles would enhance the forced convection heat transfer of water and ethanol base liquids. The rate of heat transfer enhancement is higher in fully developed region. It was observed that water based nanofluids have higher heat transfer coefficients which is related to higher thermal conductivity of water based nanofluids. Figure 15 shows variation of the heat transfer coefficient as a function of distance for ethanol and TiO_2_–ethanol nanofluids. In this figure, it was observed that man made TiO_2_ nanoparticles enhanced the heat transfer coefficient in developing region 13%, 24%, 34%, when x/D was 191, 285, and 381, respectively, also heat transfer coefficient was enhanced in fully developed region by 59%. Semiconductor TiO_2_ nanoparticles were produced in our research lab, using the dry-freezing method. It was observed that the man-made TiO_2_ nanoparticles enhanced the heat transfer coefficient of ethanol. The man-made TiO_2_ nanoparticles enhanced thermal conductivity of working fluid as well as energy transfer across the working fluid. Figure 15 compares the effects of semiconductor TiO_2_ made in our research group [40] and commercial TiO_2_ nanoparticles on the heat transfer coefficient as a function of distance. The figure shows that man-made TiO_2_ nanoparticles were enhanced the heat transfer coefficient in developing region 75%, 120%, 172%, when x/D was 334, 430, and 524, respectively, also heat transfer coefficient was enhanced in fully developed region by 185%. Similarly, commercial TiO_2_ nanoparticles were enhanced the heat transfer coefficient in developing region 36%, 90%, 119%, when x/D was 334, 430, and 524, respectively, also heat transfer coefficient was enhanced in fully developed region by 106%. For a given base liquid, it was observed that semiconductor TiO_2_ nanoparticles made in our research group [40] enhanced the forced convection heat transfer coefficient more which is related to a better and uniform distribution of man-made TiO_2_ nanoparticles in ethanol base liquid compare to commercial nanoparticles. Figure 16 shows the variation of forced convection heat transfer coefficient as a function of distance for water-TiO_2_ nanofluids with different crystal phases. The figure shows that rutile TiO_2_ nanoparticles were enhanced the heat transfer coefficient in developing region 7%, 9%, 10%, when x/D was 239, 381, and 524, respectively, also heat transfer coefficient was enhanced in fully developed region by 9%. Similarly, anatase TiO_2_ nanoparticles were enhanced the heat transfer coefficient in developing region 31%, 37%, 47%, when x/D was 239, 381, and 524, respectively, also heat transfer coefficient was enhanced in fully developed region by 36%. The rutile nanoparticles have higher electron transport property and anatase nanoparticles has a higher affinity for base liquid and therefore anatase nanoparticles mix better with base liquid compare to the rutile nanoparticles. As results, the anatase–water nanofluid has higher heat transfer coefficient. As the uniformity of the nanoparticles in the base liquid increases, the possibility of nanoparticle–nanoparticle and nanoparticle–molecule collisions and interactions increase and as a result, the energy transportation across the working fluid increases and consequently would increase the thermal conductivity [8,38] and heat transfer coefficient. Rutile TiO_2_ nanoparticles were used to enhance the efficiency of dye-sensitized and perovskite solar cells which can be seen in references [42,43,48,49,50,51,52,53,54,55,56]. The high electron transport property of rutile TiO_2_ nanoparticles discussed and reported in different references [57,58] in detail. Figure 17 shows the variation of forced convection heat transfer coefficient as a function of distance for Al_2_O_3_–water nanofluids with different nanoparticle size in range of 5 nm to 200 nm. The size of these commercial nanoparticles was given by the producer. This figure explains that the changing nanoparticle size can increase or decrease the forced convection heat transfer coefficient inside the channel. When the size of the nanoparticles is changed several other fluid factors will also change. The viscosity of the fluid, its thermal conductivity, how well the nanoparticles disperse in the liquid, and how those nanoparticles will move and collide will all be affected. The physical properties of viscosity and thermal conductivity in particular impact the forced convection heat transfer. However, these factors can contradict each other in their effects. Therefore, how the heat transfer coefficient will change depends on which factor has the greater influence based on the conditions of the fluid. The effects of nano materials on heat transfer coefficient in turbulent flow can be seen in references [41,59,60,61].

## 4. Conclusions

The effects of nanoparticles and base liquids on the forced convection heat transfer coefficient inside a stainless-steel microchannel were investigated. Specifically, Al_2_O_3_ and TiO_2_ nanoparticles were used in conjunction with ethanol and water base liquids. It was observed that the nanoparticles will enhance the forced convection heat transfer coefficient. It was also observed that water based nanofluids have higher heat transfer coefficients compared to those of ethanol based nanofluids, which might be related to the higher thermal conductivity of water.

The effects of semiconductor man-made TiO_2_ and commercial TiO_2_ nanoparticles on heat transfer coefficient was examined and it was observed that for the ethanol base liquid, semiconductor TiO_2_ nanoparticles made in our research group [40] enhanced the forced convection heat transfer coefficient more which is related to a better and uniform distribution of man-made TiO_2_ nanoparticles in base liquid compare to commercial nanoparticles.

Decreasing the nanoparticle size may decrease or increase the nanofluid thermal conductivity, however it was observed that in most cases, nanofluid thermal conductivity [8,38] and nanofluid viscosity [39] increase with decreasing nanoparticle size. It was observed that the heat transfer coefficient increases with increasing thermal conductivity and decreases with increasing viscosity. Random motion of nanoparticles and molecules decrease with increasing of viscosity; consequently, the heat transfer coefficient decreases. Therefore, decreasing nanoparticle size may decrease or increase the heat transfer coefficient, depending on which effect is dominated. If the effect of nanoparticle size on thermal conductivity is dominated, heat transfer coefficient would increase; otherwise, the heat transfer coefficient would decrease.

## Figures and Tables

**Table 1:** Summary of nanoparticles.

Nanoparticle Material	Source	Shape	Size (APS)	Weight %	Surfactant
Anatase TiO_2_	Custom ^1^	Spherical	~5–10 nm	1%	N/A
Rutile TiO_2_	Custom ^1^	Nanorods	5 nm ^3^/20 nm ^4^	1%	N/A
Anatase TiO_2_	Stock US3838 ^2^	Nearly spherical	5 nm	1%	N/A
Al_2_O_3_	Stock US3007 ^2^	Nearly spherical	5 nm	1%	N/A
Al_2_O_3_	Stock US3008 ^2^	Rhombohedral	80 nm	1%	N/A
Al_2_O_3_	Stock US3002 ^2^	Rhombohedral	200 nm	1%	N/A

^1^ Particle synthesis process explained below, ^2^ US Research Nanomaterials, Inc. (Houston, TX, USA), ^3^ Diameter, ^4^ Length.

**Table 2:** Summary of nanofluid properties.

Material	Density [kg/m^3^]	Specific Heat [J/kg-K]	Dynamic Viscosity [mPa-s]	Thermal Conductivity [W/m-K]
Nanoparticles				
TiO_2_	3900 ^1^	850	-	-
Al_2_O_3_	3970 ^1^	955	-	-
Nanofluid				
TiO_2_–water	1004.5	4153.6	0.653	0.684
Al_2_O_3_–water	1004.5	4154.7	0.600	0.673
TiO_2_–ethanol	795.34	2552.8	0.718	0.170
Al_2_O_3_–ethanol	795.37	2553.9	0.717	0.166

^1^ US Research Nanomaterials, Inc.

## Data Availability

The data presented in this study can be used for further investigation.

## Nomenclature

V%	Volume percentage (-)
wt%	Weight percentage (-)
Re	Reynolds number (-)
Nu	Nusselt number (-)
ID	Inner diameter (m)
D	Pipe diameter (m)
x	Critical length (m)
h	Heat transfer coefficient ($W/m^{2}.K$)
T	Temperature (K)
$c_{p}$	Specific heat (J/kg.K)
$\dot{m}$	Mass flow rate (kg/s)
A	Heat transfer area ($m^{2}$)
${\dot{Q}}_{absorbed}$	Rate of energy absorption (W)
$\dot{q}$	Heat flux ($W/m^{2}$)
Greek Symbols	
ϕ	Nanoparticle Volume fraction (-)
ρ	Density ($kg/m^{3}$)
Subscripts	
nf	Nanofluid
oil	Oil
bf	Base fluid
p	Particle
in	Inlet
out	Outlet

## Appendix A

**Table A1:** Summary of existing experimental investigations related to heat transfer coefficient.

Reference	Specifications	Remarks
Muryam et al. [26] (2017)	Nanofluid: Gold and silver nanoparticles were mixed with deionized water to produce gold-water and silver-water nanofluids at different nanoparticles volume fractions of 0.015 vol %, 0.045 vol %, and 0.0667 vol %. Flow regime: Laminar Heated section: 580 mm long, horizontal, straight stainless-steel tube, 2.27 mm inner diameter and 6.27 mm outer diameter. Heat transfer: The test section was resistively heated by a DC power supply to generate a constant heat flux.	The effects of the entrance region were noted for all cases. It was observed that (a) the local heat transfer coefficient and Nusselt number increased with nanoparticle volume fraction consistently and (b) the gold nanoparticles enhanced the heat transfer coefficient more than silver nanoparticles. It was explained that the nanofluid heat transfer coefficient was attributed to (a) nanofluid thermal conductivity enhancement, (b) reduction of thermal boundary layer thickness, and (c) random motion of nanoparticles. The experimental results indicated that the heat transfer coefficient of gold nanofluid was higher than silver nanofluid heat transfer coefficient due to high thermal conductivity of gold nanofluid for the given conditions.
Ebrahimnia-Bajestan et al. [4] (2016)	Nanofluid: TiO_2_ nanoparticles with a mean diameter of 21 nm dispersed into deionized water, at different nanoparticles volume concentrations of 1 vol %, 1.6 vol %, and 2.3 vol %. Flow regime: Laminar Heated section: 2 m long, horizontal, straight copper tube, 7.8 mm inner diameter, and 9.6 mm outer diameter. Heat transfer: The tube was heated by a flexible silicone rubber heater which was linked to a DC power supply to provide constant heat flux.	Laminar convective heat transfer of TiO_2_–water nanofluid flowing through a uniformly heated tube was investigated experimentally and numerically. The average heat transfer coefficient as a function of Reynolds number was compared with prediction of the Shah correlation to certify the reliability and accuracy of the experimental setup and numerical predictions. A reliable agreement between the experimental results and the theoretical prediction was observed with the maximum deviation of 3%. The maximum enhancement of the average heat transfer coefficient was 21%, using TiO_2_–water nanofluids at a low concentration of 2.3 vol % where the Reynolds number was ~940. Variation of the local heat transfer coefficient with distance for different Reynolds numbers and nanoparticle volume fractions was investigated. Variations of the average heat transfer coefficient with Reynolds number were investigated. The experimental data and the numerical prediction were compared, and good agreement was observed. In case of numerical investigations, the single-phase model was compared with the common two-phase numerical approaches. The predicted heat transfer coefficients, using single-phase and common two-phase approaches, underestimate and overestimate the experimental data, respectively. The two-phase model was modified to enhance the level of accuracy of predictions of the heat transfer characteristics of nanofluids. This modified model predicted that the convective heat transfer coefficient increased with nanoparticle concentration and flow Reynolds number, and decreased with particle size. Nanofluids with smaller nanoparticles size and higher thermal conductivity were recommended for solar liquid heating collectors.
Umer et al. [29] (2016)	Nanofluid: 20 nm Cu_2_O nanoparticles were mixed with water at volume fraction of 1 vol %, 2 vol %, and 4 vol %. Flow regime: Laminar Heated section: Straight stainless-steel tube Heat transfer: The stainless-steel tube was heated by passing current through the pipe to generate a constant heat flux.	Variation of local heat transfer coefficient with distance for different Reynolds numbers and volume fractions were investigated. It was observed that the heat transfer coefficient increased with volume concentration and Reynolds number. The maximum heat transfer coefficient enhancement was 61% where the particle volume concentration was 4% and Reynolds number was ~605. The variation of the local heat transfer coefficient with distance was compared with the theoretical prediction and good agreement was observed.
Akhavan-Zanjani et al. [16] (2016)	Nanofluid: Graphene-water nanofluid at volume fraction of 0.005 vol %, 0.01 vol %, and 0.02 vol %. Composite graphene sheets approximately 1.4–2.3 nm thick. Flow regime: Laminar, 600<Re<1850 Heated section: A straight copper tube with inner diameter of 4.20 mm and outer diameter of 6 mm and length of 2740.2 mm. Heat transfer: To apply the uniform wall heat flux boundary condition, the tube surface in the test section was uniformly heated by nickel–chrome wire which was uniformly wound around the tube and linked by an AC power supply.	Variation of the local heat transfer coefficient with distance for different nanoparticle volume fraction was investigated. Variation of the average Nusselt number with Reynolds number for different volume fraction was investigated. Adding a small amount (up to 0.02% volume fraction) of graphene nanoparticles into water increased the thermal conductivity and convective heat transfer coefficient of the working fluid significantly. The maximum enhancement was 10.3% for thermal conductivity and 14.2% for the heat transfer coefficient at Re = 1850.
Noghrehabadi and Pourrajab [15] (2016)	Nanofluid: 20 nm spherical γ-Al_2_O_3_ nanoparticles were mixed with water to produce nanofluid at volume fraction of 0.1 vol %, 0.3 vol %, and 0.9 vol %. Flow regime: Laminar, 1057<Re<2070 Heated section: A straight copper tube with inner diameter of 11.10 mm and thickness of 1.6 mm and length of 2380 mm. Heat transfer: The copper was electrically heated by nichrome wire, uniformly wound on the tube and connected to an AC power supply to provide a constant heat flux.	The local heat transfer coefficient was measured as a function of distance from the entrance for different volume fractions and different Reynolds numbers in laminar flow where the inlet temperature was 30 °C. The maximum heat transfer enhancement was 23% where the volume fraction was 0.9%. The nanofluid heat transfer coefficient increased with volume fraction. The Shah equation could not predict the experimental data, therefore a new correlation for alumina-water nanofluids was created using curve fitting with mean deviation of 3.57% from the experimental data. $Nu_{x}=4.36+\left(3+\varphi^{0.442}\right)Re_{nf}^{0.288}Pr_{nf}^{0.0185}{\left(\frac{d}{x}\right)}^{0.3851}$ 0.1%<φ<0.9% 900<Re<2100 40<x/D<180
Cabaleiro et al. [25] (2015)	Nanofluid: Homogeneous and stable nanofluids were prepared by dispersing dry zinc oxide, ZnO (40–100 nm), nanoparticles at mass concentrations of 1 wt %, 2.5 wt %, and 5 wt % in ethylene glycol + water mixtures, at 50/50% in volume. Flow regime: Laminar and transition from laminar to turbulent Heated section: 2 m length straight copper pipe with an inner diameter of 8 mm and an outer diameter of 12 mm. Heat transfer: The heating electrical resistance was continuously wound around the copper pipe to achieve a uniform heat flux boundary condition along the test section.	In a mixture of ZnO, water and ethylene glycol, the direct contact between surfactant-free ZnO nanoparticles and water was prevented by the ethylene glycol presence around ZnO nanoparticles. No enhancement in heat transfer coefficient was observed. A good agreement was also found between the experimental heat transfer coefficient and theoretical predictions.
Heris et al. [3] (2015)	Nanofluid: Different nanofluids were made including CuO-turbine oil (0.1 vol %, 0.2 vol %, 0.4 vol %, and 0.5 vol %), TiO_2_-turbine oil (0.1 vol %, 0.25 vol %, 0.35 vol %, and 0.5 vol %) and Al_2_O_3_-turbine oil (0.1 vol %, 0.3 vol %, 0.4 vol %, and 0.5 vol %). CuO, Al_2_O_3_, and TiO_2_ nanoparticles had 30 nm, 50 nm and 30 nm sizes, respectively. Flow regime: Laminar Heated section: A copper tube with 1.3 m in length, 8 mm in outer diameter and 7 mm in inner diameter. Heat transfer: The electrical resistance and DC power supply were used to obtain a constant heat flux condition.	Variation of the local heat transfer coefficient with distance for different nanoparticle volume fractions was investigated. Variation of the Nusselt number with Reynolds number for different nanoparticle volume fractions was investigated. The experimental data was compared with the Sieder-Tate prediction. It was observed the heat transfer coefficient was much higher than the prediction. CuO-turbine oil nanofluid showed the highest heat transfer coefficient at 0.5 vol % nanoparticle volume fraction. It was concluded that even though the thermal conductivity enhancement of nanofluids has a key role in the enhancement of the heat transfer coefficient it was not the only responsible factor for the modification of the heat transfer coefficient and there must have been different mechanisms involved.
Minakov et al. [32] (2015)	Nanofluid: CuO nanoparticles were mixed with water to produce nanofluid with 0.25 vol %, 0.5 vol %, 1 vol %, and 2 vol % nanoparticle volume fractions. The size of CuO nanoparticles was 30 nm. Flow regime: Laminar Heated section: A copper tube with length of 1 m, outer diameter of 6 mm and inner diameter of 4 mm. Heat transfer: A 0.1 mm thick nichrome wire with total resistance of 320 Ω wound around the channel was used as a heater.	The local heat transfer coefficient was measured as a function of distance for the entrance region. The local heat transfer coefficient increased with volume fraction. For instance, 2 vol % CuO-water nanofluid enhanced the local heat transfer about 12%, along the entire channel. The average heat transfer coefficient was measured as a function of mass flow rate and Reynolds number. The average heat transfer coefficient increased with mass flow rate. The concentration of nanoparticles had a significant influence on the dependency of the average heat transfer coefficient to flow rate. For instance, for 2 vol % nanoparticle concentration and a given flow rate the heat transfer coefficient was about 13% higher than that for water. Similarly, for 2 vol % nanoparticle concentration and a given Reynolds number the heat transfer coefficient was more than 40% higher than that for water. Moreover, for 0.25 vol % nanoparticle concentration the laminar-turbulent transition was slightly delayed, while the laminar-turbulent transition happened for pure water when the Reynolds number was over 2000 as it was expected.
Esmaeilzadeh et al. [35] (2014)	Nanofluid: Al_2_O_3_–water nanofluid was prepared by dispersing γ-Al_2_O_3_ nanoparticles in distilled water. The average nanoparticle size was 15 nm. The volume concentrations were 0.5 vol % and 1 vol %. Flow regime: Laminar, 150<Re<1600 Heated section: A twisted copper tape inserted into a copper tube with length, inner and outer diameters of 1000 mm, 7.0035 mm, and 9 mm, respectively. The twisted tape was made by twisting copper strip of 1000 mm length, various thicknesses (0.5 mm, 1 mm, and 2 mm) with twist ratio of 3.41. Heat transfer: The electrical resistance (nickel chrome) and AC power supply were used to obtain a constant heat flux condition.	The Nusselt number was measured as a function of distance from the entrance for pure water, Re = 919.3, and compared with the prediction of the Shah correlation. The deviation of experimental Nusselt number and correlation was in range of 0.1–5.1%. The Nusselt number as a function of Reynolds number and the local heat transfer coefficient as a function of distance were measured for different twisted tape thicknesses and for different working fluids such as water and alumina nanofluids (0.5 vol % and 1 vol %). It was found that (a) the heat transfer coefficient increased with the thickness of inserted twisted tape, the result indicated that the pure water heat transfer coefficient enhancement were 75.03%, 80.20%, and 90.58% respectively for twisted tape thicknesses of 0.5 mm, 1 mm, and 2 mm; (b) the maximum heat transfer coefficient enhancement occurred at the pipe entrance and it reduced with axial distance in entrance region; and (c) the heat transfer coefficient enhancement increased with nanoparticle volume fraction. A new correlation was developed to predict the Nusselt number when twisted tape was inserted into the tube. The new correlation had a maximum deviation of 15% from the experimental data.
Kumaresan et al. [41] (2013)	Nanofluid: Multi-wall carbon nanotubes (MWCNT) dispersed in water–ethylene glycol mixture (70:30 by volume). Base liquid was a mixture of DI-water 70 vol % and ethylene glycol (EG) 30 vol % and sodium dodecyl benzene sulphonate (SDBS) 0.1 vol % as a surfactant. The volume fractions were 0.15 vol %, 0.30 vol %, and 0.45 vol %. The nominal average diameter of MWCNT was 30–50 nm and length of 10–20 μm. Flow regime: Turbulent, 4000<Re<20000 Heated section: A 2.5 m long counter flow concentric tubular heat exchanger, in which the nanofluid flows through the inner smooth copper tube with an inner diameter of 10.7 mm while water flows through the outer tube with an outer diameter of 25.4 mm.	The local heat transfer coefficient was measured as a function of distance from the entrance for different particle volume fractions, Reynolds numbers, and nanofluid velocities. It was observed that local heat transfer coefficient increased with MWCNT volume fraction. The Nusselt number was measured as a function of Reynolds number for different MWCNT volume fractions. The Nusselt number increased with Reynolds number and the rate of this enhancement increased with MWCNT volume fraction. It was suggested that the migration of carbon nanotubes could be the possible reason for nanofluid heat transfer enhancement at the entrance region. The pure water experimental data were compared with the prediction of the Shah correlation for laminar heat transfer and the Gnielinski correlation for turbulent heat transfer. The experimental values were in acceptable agreement with the predicted values for laminar and turbulent flow regimes for pure water. However, they could not predict the nanofluid heat transfer for nanofluids.
Torii and Yang [59] (2009)	Nanofluid: Nano-diamonds-water (0.1 vol %, 0.4 vol %, and 1 vol %). The nanoparticle was in size of 2 nm to 10 nm. Flow regime: Turbulent, Re = 3000–6000 Heated section: A straight stainless-steel tubing with an inner diameter (ID) of 4 mm, outer diameter (OD) of 4.3 mm, and length of 1 m. Heat transfer: The test section was resistively heated by a DC power supply to generate a constant heat flux.	The local heat transfer coefficient was measured as a function of distance from the entrance for different nanoparticle volume fractions in turbulent flow. It was observed that (a) the heat transfer coefficient increased with nano-diamond volume fractions, (b) the heat transfer coefficient decreased with distance, for a given volume fraction. The local heat transfer coefficient approached a constant value along the axial direction. The Nusselt number as a function of Reynolds number was measured for different volume fractions at x/D = 180. The Nusselt number increased with nanoparticle volume fraction for a given Reynolds number at x/D = 180. The experimental data was compared with the prediction of Gnielinski’s correlation. Good agreement was observed between the prediction and the experimental data for pure water and low concentration nanofluids. It was also suggested that substantial heat transfer coefficient enhancement was not attributed purely to the nanofluid thermal conductivity enhancement.
Liu and Yu [17] (2011)	Nanofluid: The alumina nanofluid was prepared by dispersing commercial 40 nm, γ- phase Al_2_O_3_ nanoparticles in deionized water. The nanoparticle volume fractions were 1 vol %, 2 vol %, 3.5 vol %, and 5 vol %. Flow regime: The Reynolds number varied from 600 to 4500, covering the laminar, transition, and early fully developed turbulent regions. Heated section: The test tube was a circular mini-channel made of stainless steel, where inner diameter was 1.09 mm and outer diameter was 1.34 mm. The total length was 306 mm. Heat transfer: The test section was resistively heated by a DC power supply to generate a constant heat flux.	In laminar region, the measured Nusselt number of pure water was in good agreement with the prediction from the Oskay–Kakac correlation In the turbulent region, the Hausen correlation for thermally developing turbulent flow was the best in predicting the measured Nusselt number of pure water. The heat transfer coefficient was measured as a function distance for two different Reynolds numbers, Re = 870 and 1230. The nanofluids heat transfer coefficient was enhanced with Reynolds number and the nanoparticle concentration. The heat transfer enhancement was more significant in the entrance region than at downstream locations. For the 5 vol % nanofluid, the heat transfer coefficient enhancement was 19% near the entrance and decreased to less than 9% near the channel exit. In laminar flow, the average nanofluid Nusselt number was measured and experimental data were bounded by the predictions of the Oskay–Kakac correlation and the Stephan correlation, whereas the Hausen equation for laminar flow almost under predicted the measured data. In the transition and turbulent regions, the average nanofluid Nusselt numbers were measured. As Re increased, there were two locations where the slope of the experimental data changed. The first one corresponded to the onset of transition to turbulence, and the second one was associated with the beginning of fully developed turbulent heat transfer. It can be observed that the delayed transition occurred at Re_cr_ = 2800. The average Nusselt number was measured for the nanofluids and the base fluid over the entire range of Re, from laminar to fully developed turbulent flow. It was found that: (a) nanofluids enhanced convective heat transfer moderately in laminar flow, (b) nanofluids caused significant heat transfer deterioration in the transition and the early stage of fully developed turbulent regions, (c) both the enhancement and deterioration were seen to increase with the nanoparticle concentration, (d) for nanofluids, onset of transition to turbulence was delayed, (e) once the flow becomes fully turbulent, the difference in the measured Nusselt number between nanofluids and water diminished. Established conventional correlations cannot fully predict the heat transfer of nanofluids, particularly in the transition and turbulent regions, even when the effective thermophysical properties are taken into consideration. The results from this work suggest that the particle–fluid interaction has a significant impact on the flow physics of nanofluids, especially in the transition and turbulent regions.
Duangthongsuk and Wongwises [60] (2010)	Nanofluid: TiO_2_–water nanofluids with 0.2 vol %, 0.6 vol %, 1.0 vol %, 1.5 vol %, and 2 vol % volume fractions were used. The nanoparticles were 21 nm in size. Flow regime: Turbulent, Re = 3000–18,000 Heated section: A 1.5 m long counter-flow horizontal double tube heat exchanger with nanofluid flowing inside the tube while hot water flows in the annular. The inner tube was made from smooth copper tubing with 9.53 mm outer diameter and 8.13 mm inner diameter.	Heat transfer coefficient and Nusselt number was measured as a function of Reynolds number for different nanoparticle volume fractions. The heat transfer coefficient and Nusselt number increased with Reynolds number, however the effects of the nanoparticle volume fraction on heat transfer coefficient was not consistent. For φ<1.0%, the heat transfer coefficient increased with increasing volume fraction, compared with the pure base liquid. At 1 vol %, the enhancement range was between 20% and 32%. The heat transfer coefficient at φ=1.5% was smaller than that of at φ=1.0% and the heat transfer coefficient at φ=1.5% was larger than that of the base liquid. The heat transfer coefficient of nanofluid at φ=2% was nearly 14% less than that of pure water, for the given conditions. The measured Nusselt number was compared with the predicted Nusselt number by the Gnielinski correlation. A reasonable agreement was observed for pure water. Similarly, the measured Nusselt number was compared with the predicted Nusselt number by Pak and Cho [61] for nanofluids with different volume fractions and no agreement was observed. A correlation was developed, using curve fitting method to predict the Nusselt number as follows $Nu_{nf}=0.074Re^{0.707}Pr^{0.285}\varphi^{0.074}$ The predicted Nusselt number was plotted against the experimental Nusselt number and it was observed the majority of the data falls within ±10% of the proposed equation.
Anoop et al. [37] (2009)	Nanofluid: Alumina-water (1 wt %, 2 wt %, 4 wt %, and 6 wt %). The nanoparticles were 45 nm and 150 nm in size. Flow regime: Laminar, Re = 500–2000 Heated section: A straight copper tubing with an inner diameter (ID) of 4.75 mm, outer diameter (OD) of 7.25 mm, and length of 1.2 m. Heat transfer: Electrically insulated nickel chrome wire was uniformly wound along the length to heat up the tube. A DC power supply was used as a power source.	The local nanofluid heat transfer coefficient as a function of Reynolds number was measured for different nanoparticle volume fractions and two different nanoparticle sizes, 45 nm and 150 nm. It was found that (a) the nanofluid heat transfer coefficient increased with increasing Reynolds number and nanoparticle volume fraction; and (b) at lower x/D, the heat transfer coefficient changed with Reynolds number but at x/D > 200, the nanofluid heat transfer coefficient was independent of Reynolds number. The heat transfer coefficient increased with decreasing nanoparticle size. For instance, for x/D = 147, Re = 1550, 45 nm nanoparticle size, the nanofluid heat transfer coefficient enhancement was ~25% whereas the thermal conductivity for same conditions increased only by ~6%. As particle size was reduced the thermal conductivity of the nanofluid increased. Likewise, for 150 nm nanoparticle size, the nanofluid heat transfer coefficient enhancement was ~11% whereas the thermal conductivity increased ~4% only for almost similar conditions. They speculated that thermal conductivity enhancement may not be the only reason for heat transfer coefficient enhancement. The enhancement took place mostly in the entrance region than at a higher x/D. For instance, at ~Re = 1550, the heat transfer coefficient enhancement was 31%, 25%, and 10% respectively at x/D = 63, 147, and 244. A correlation was developed as: $Nu_{x}=4.36+\left[ax_{+}^{-b}\left(1+\varphi^{c}\right)exp\left(-dx_{+}\right)\right]\left[1+e{\left(\frac{d_{p}}{d_{ref}}\right)}^{-f}\right]$ $a=6.219*10^{-3},\;b=1.1522,\;c=0.1533,\;d=2.5228,\;e=0.57825,\;f=0.2183,\;d_{ref}=100\;\mathrm{nm}$ $x_{+}=\frac{x}{DRePr}$ $d_{p}$ was diameter of particle The predicted Nusselt number was plotted against of experimental Nusselt number, and the correlation fell in the region of ±20% deviation.
Rea et al. [14] (2009)	Nanofluid: Alumina/water (0.6 vol %, 1 vol %, 3 vol %, 6 vol %) and zirconia/water (0.32 vol %, 0.64 vol %, 1.32 vol %). The nanoparticle size was 50 nm for both alumina and zirconia. Flow regime: Laminar, Re = 140–1888 Heated section: Vertical stainless-steel tubing with an inner diameter (ID) of 4.5 mm, outer diameter (OD) of 6.4 mm, and length of 1.01 m. Heat transfer: The test section was resistively heated by a DC power supply to generate a constant heat flux.	Laminar convective heat transfer investigated for alumina–water and zirconia–water nanofluids as a function of distance in a vertical heated tube. The heat transfer coefficients in the entrance region and in the fully developed region were found to increase by 17% and 27%, respectively, for alumina–water nanofluid at 6 vol % with respect to pure water. The zirconia–water nanofluid heat transfer coefficient increased by approximately 2% in the entrance region and 3% in the fully developed region at 1.32 vol %. The nanofluids can be treated as homogeneous mixtures. The alumina nanofluid Nusselt number as a function of distance for the four different volumetric loadings (0.6%, 1%, 3%, and 6%) were plotted. The Nusselt numbers were in good agreement with the theoretical prediction. The zirconia nanofluid Nusselt number as a function of distance for three volumetric loadings (0.32%, 0.64%, and 1.32%) were plotted. The Nusselt numbers were in good agreement with the theoretical prediction.
Williams et al. [22] (2008)	Nanofluid: Alumina/water (0.9 vol %, 1.8 vol %, and 3.6 vol %) and zirconia/water (0.2 vol %, 0.5 vol %, and 0.9 vol %). The average particle size for the alumina was about 46 nm and for the zirconia was about 60 nm. Flow regime: Turbulent, 9000<Re<63000 Heated section:Horizontal stainless-steel tubing with an outer diameter (OD) of 12.7 mm, and thickness of 1.65 mm. Heat transfer: The test section was resistively heated by a DC power supply to generate a constant heat flux.	The nanoparticle concentrations were 0.9–3.6 vol % and 0.2–0.9 vol % for alumina/water nanofluid and zirconia/water nanofluid respectively. The experimental data was compared to the theoretical predictions and good agreement was observed. The most interesting finding was that the convective heat transfer of the alumina/water and zirconia/water nanofluids tested in fully developed turbulent flow can be predicted by the traditional correlations and models, as long as the effective nanofluid physical properties were used.
Chen et al. [19] (2008)	Nanofluid: Titanate nanotube mixed with water to produce nanofluid with nanotube volume fraction of 0.12 vol %, 0.24 vol %, and 0.6 vol %. The aspect ratio was ~10 (10 nm diameter and 100 nm length). Flow regime: Laminar, 1100<Re<2300 Heated section: A straight copper tubing with an inner diameter (ID) of 3.97 mm, outer diameter (OD) of 6.35 mm, and length of 2 m. Heat transfer: The test section was heated by two flexible silicone rubber heaters, linked to a DC power supply.	The local heat transfer coefficient as a function of distance for different nanoparticle mass fractions was measured. An excellent heat transfer enhancement was observed despite the small thermal conduction enhancement. Likewise, the local heat transfer coefficient as a function of distance was measured for different Reynolds numbers. It was observed the heat transfer coefficient increased with Reynolds number for all cases. For similar conditions, the enhancement of both the thermal conductivity and the convective heat transfer coefficient of the titanate nanotube nanofluids were considerably higher than those of spherical titanate nanoparticles which indicated the important role of particle shape in heat transfer enhancement.
Wen and Ding [30] (2004)	Nanofluid: 27–56 nm alumina-water (0.6 vol %, 1 vol %, and 1.6 vol %). Flow regime: Laminar, 500<Re<2100 Heated section: A straight copper tube with 970 mm length, 4.5 mm inner diameter, and 6.4 mm outer diameter was used. Heat transfer: The test section was heated by a flexible silicone rubber heater to provide constant heat flux.	The local heat transfer coefficients as a function of the axial distance for different nanoparticle volume fractions in Re = 1050 and 1600 were measured. The results indicated that nanofluid significantly enhanced the heat transfer coefficient, particularly at the entrance region at higher Reynolds numbers. It was found that the thermal developing length of nanofluids was greater than that of the base liquid which increased with increase of particle concentration. Likewise, the Nusselt number was measured against the Reynolds number for different nanoparticle volume fractions at laminar flow. It was observed the nanofluid Nusselt number was higher than that of water and the Shah equation failed to predict the nanofluid experimental data. It was proposed that the heat transfer coefficient enhancement was because of the thermal conductivity enhancement as well as particle migration.
